# Integration of Multiple-Omics Data to Analyze the Population-Specific Differences for Coronary Artery Disease

**DOI:** 10.1155/2021/7036592

**Published:** 2021-08-17

**Authors:** Yang Hu, Shizheng Qiu, Liang Cheng

**Affiliations:** ^1^Department of Computer Science and Technology, School of Life Science and Technology, Harbin Institute of Technology, Harbin, China; ^2^College of Bioinformatics Science and Technology, Harbin Medical University, Harbin, China

## Abstract

Significant differences may exist among different descents, but the current studies are mainly based on European populations. In the present study, we analyzed the population-specific differences of coronary artery disease (CAD) between European and East Asian descents. In stage 1, we identified CAD susceptibility genes by gene-based tests in European and East Asian populations. We identified two novel susceptibility genes for CAD, namely, *CUX2* and *OAS3*. In stage 2, we carried out meta-analyses for the population-specific variants. rs599839 (*PSRC1*) represented a protective variant for CAD in East Asian populations (OR_ASN_ = 0.72. 95% CI: 0.63-0.81) but a risk factor in European populations (OR_EUR_ = 1.13, 95% CI: 0.93-1.36). In stage 3, we enriched the risk genes and explored the population-specific differences in Gene Ontology (GO), Kyoto Encyclopedia of Genes and Genomes (KEGG), regulatory element, tissues, and cell types. In stage 4, in order to predict genes that showed pleiotropic/potentially causal association with CAD, we integrated summary-level data from independent genome-wide association studies (GWAS) and expression quantitative trait loci (eQTLs) by using summary data-based Mendelian randomization (SMR). The results showed that *NBEAL1* and *FGD6* were population-specific pleiotropic/causal genes. Although some potential mutations and risk genes of CAD are shared, it is still of great significance to elucidate the genetic differences among different populations. Our analysis provides a better understanding of the pathogenic mechanisms and potential therapeutic targets for CAD.

## 1. Introduction

Coronary artery disease (CAD) remains one of the leading causes of mortality worldwide [1, 2]. Although many efforts have been made to prevent and treat CAD, there is still a long way to go to curb the development of CAD, especially in underdeveloped countries and regions [3]. Epidemiological studies have shown that the occurrence of CAD is caused by both genetic and environmental factors, with gender, age, smoking, drinking, hypertension, dyslipidemia, diabetes, obesity, and mental stress being its potential risk factors [4].

Thanks to genome-wide association studies (GWAS), more than one hundred and sixty CAD susceptibility loci have been identified [5–8]. However, some drawbacks exist using GWAS to identify susceptibility loci. First of all, GWAS only reported the genetic variants significantly correlated with the trait (*P* < 5*E* − 08) but seldom considered the variants moderately correlated or uncorrelated with the trait. Secondly, more than 90% of the variants identified by GWAS are located in noncoding regions (introns or intergenic regions) [9, 10]. The function of variants in the regulatory region is still unclear. Thirdly, due to the complex linkage disequilibrium (LD) between pathogenic mutation sites and other SNPs, the genes closest to the lead SNPs in the physical distance are not necessarily the most likely causal genes [11–13]. Finally, GWAS explain only a modest fraction of the missing heritability of human diseases [14, 15]. Therefore, it is far from enough to analyze the risk loci from lead SNPs alone.

Furthermore, significant differences have been found in the risk of CAD among different descents, but the current GWAS is mainly based on European populations [16]. A deeper understanding of the genetic structure of other ethnic groups will lead to the development of drugs for population-specific targets and the precise treatment of patients. Previous studies have used the polygenic risk score (PRS) to assess the differences in clinical risk factors for CAD between Japanese and European populations and found several novel loci [17, 18]. However, European ancestry GWAS from the CARDIoGRAMplusC4D 1000 Genomes meta-analysis of previous studies contains some Asian, Hispanic, and African ancestry, which may confuse population-specific loci [8, 18].

In this study, we used two large-scale GWAS which were made up entirely of European and Japanese ancestry, respectively, to identify risk loci by gene-based tests. By integrating GWAS and eQTL datasets, we comprehensively analyzed population-specific differences of susceptibility loci, genetic variants, tissue, cell lines, regulatory elements, and metabolic pathways. Our study provides new insights into the heterogeneity of CAD in the stratification of different populations.

## 2. Materials and Methods

### 2.1. Data Sources

Two large-scale GWAS and 54 eQTLs were used in this study. European ancestry GWAS was obtained from a meta-analysis of 14 GWAS of CAD comprising 22,233 cases and 64,762 controls by the CARDIoGRAMplusC4D Consortium [6]. East Asian ancestry GWAS was obtained from the GWAS Catalog which included 2,808 cases and 7,261 controls [18, 19]. Both of them were each made up of a single population. The corresponding SNPs, effect allele, other allele, effect allele frequency (EAF), beta coefficients (*β*), standard errors (se), *P* value, and sample numbers were obtained from the above two datasets. We selected 54 eQTL summary data from the Genotype-Tissue Expression (GTEx, version 7) [13, 20, 21]. The donors were of multiple descents including European (85.3%), African (12.3%), and Asian (1.4%) [21]. All data used in this study are allowed to be available in the public database.

### 2.2. Identification of Susceptibility Genes

Gene-based association analyses were used to identify risk genes [22]. Compared with the traditional approach based on genome-wide association (*P* < 5*E* − 08), the gene-based approach considers the association of traits with all SNPs. Several SNPs in a gene may not be highly associated with traits but may play an important role in traits together. Herein, we defined gene boundaries in this case as ±50 kb of 5′ and 3′UTRs and used VEGAS to calculate the risk statistics of CAD for each gene in GWAS [23]. VEGAS combined information from a full set of SNPs (markers) within a gene and accounts for LD between SNPs by using simulations from the multivariate normal distribution [23].

### 2.3. Meta-Analysis of Population-Specific Genetic Variants

Polymorphisms of genetic variants were associated with CAD risk. In order to explore population-specific variants, we pooled the published population-specific variants from CAD GWAS with the variants we used the gene-based test to identify. We then reviewed above susceptibility variants in CAD case-control studies in NCBI PubMed. Finally, we carried out meta-analysis and sensitivity tests. Considering the heterogeneity between studies, the *I*^2^ statistic was used to evaluate the heterogeneity, and the magnitude of the variation was determined using *τ*^2^ [24]. In cases where *I*^2^ is greater than 50% and funnel plots were asymmetric, we tended to use random-effects models to combine effect values. The criteria for selecting study variants were that the total number of studies at the variant was greater than five and that *β* (OR), se, *P* value, and genetic models were disclosed clearly. The details of studies that met the criteria for meta-analysis are shown in Supplementary Table 1 and Supplementary Table 2.

### 2.4. Pathway, Regulatory Element, Tissue, and Cell-Type Enrichment Analyses

For susceptibility loci that passed the risk statistics threshold (*P* < 0.01), we first used hypergeometric distribution test to evaluate whether CAD risk genes (protein-coding genes) were significantly enriched in GO terms and the KEGG pathway [25]. Secondly, in order to test the relationship between highly expressed genes and genetic associations in a specific tissue, gene-property analysis was performed using the average expression of genes per tissue type by MAGMA [26–28]. Thirdly, a gene expression heat map was used to indicate the expression of susceptibility loci in 54 human tissues of GTEx, and the SciPy package of Python helped with displaying hierarchical clustering [21, 28]. Finally, we used GARFIELD (GWAS Analysis of Regulatory or Functional Information Enrichment with LD correction) to enrich the elements in the regulatory region [29]. We assessed the enrichment of association analysis signals in 1005 features extracted from ENCODE, GENCODE, and Roadmap Epigenomics projects, including histone modifications, DNase I hypersensitive sites (DHSs), and transcription factor binding sites [9, 10, 29, 30].

### 2.5. Identification of Causal Gene Targets

In order to identify genes that showed pleiotropic/causal association with CAD, we integrated summary-level data from independent GWAS and eQTLs to perform summary data-based Mendelian randomization (SMR) [13]. SMR was based on the framework of Mendelian randomization (MR), which could determine whether gene expression (exposure) was related to traits (outcome) [13, 31, 32]. Therefore, the effect of gene expression (*x*) on trait (*y*) can be expressed as the ratio of the least-square estimates of *y* and *x* on a genetic variant (*z*), respectively, namely,

(1) $$\begin{array}{c} \hat{b_{xy}}=\frac{\hat{b_{zy}}}{\hat{\beta_{zx}}}. \end{array}$$

In order to test the significance of *b*_*xy*_, the *T* statistic was designed as

(2) $$\begin{array}{c} T_{\text{SMR}}=\frac{\hat{b_{xy}^{2}}}{\mathrm{var}\left(\hat{b_{xy}}\right)}, \end{array}$$

where $\mathrm{var}\left(\hat{b_{xy}}\right)$ was the sampling variance of the two-step least-square (2SLS) estimate of *b*_*xy*_ and *T*_SMR_ was a statistic of the approximate *χ*^2^ test. For the variants that passed the test threshold, three possible explanations for the association between a trait and gene expression existed, including pleiotropy, causality, and linkage. Since the biological significance of linkage genes might lack value, we used heterogeneity in dependent instruments (HEIDI) to distinguish functional association from linkage subsequently [13]. Rejection of the null hypothesis (*P*_HEIDI_ < 0.05) indicated that the observed association might be due to two distinct genetic variants in high LD [33]. For each probe in eQTLs, only the top associated cis-eQTL was used as the instrument for the SMR test. Eventually, we analyzed common and specific CAD gene targets in different populations and pinpointed functionally relevant genes adjacent to it on the chromosome.

## 3. Results

### 3.1. Novel Susceptibility Loci for CAD

**Figure 1:**
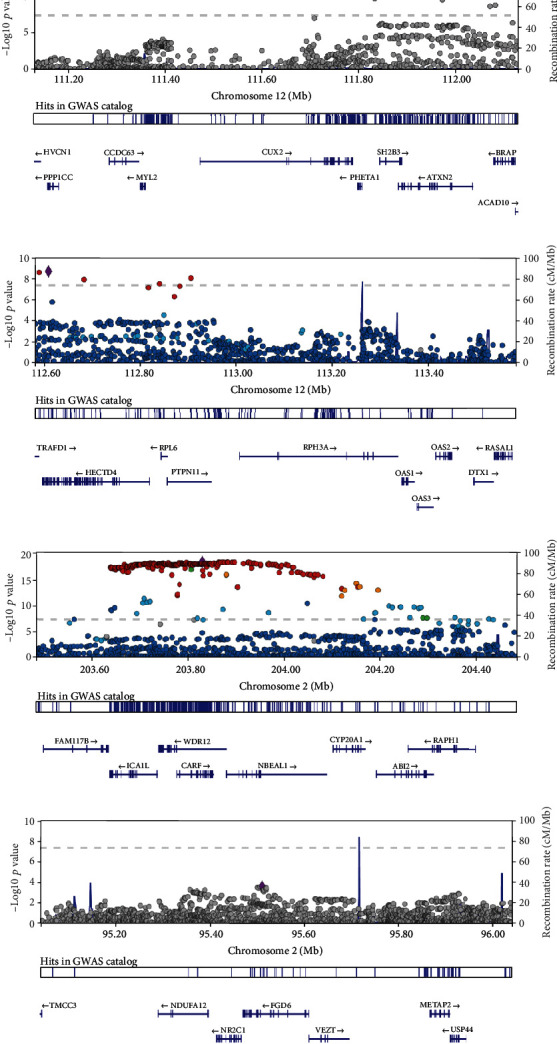
LocusZoom plots for novel loci. The *x*-axis denotes chromosomal location and the *y*-axis denotes -log_10_*P* value for each SNP. Variants in linkage disequilibrium with the lead variant are shown in red (0.8 < *r*^2^ ≤ 1), orange (0.6 < *r*^2^ ≤ 0.8), green (0.4 < *r*^2^ ≤ 0.6), sky blue (0.2 < *r*^2^ ≤ 0.4), light blue (0 < *r*^2^ ≤ 0.2), and gray (no *r*^2^ data).

In contrast to the 1000 Genome Project (1KGP) reference panels, LD between SNPs were calculated [34]. By carrying out a gene-based test, 12 and 42 susceptibility genes for CAD passed the FDR threshold (0.05/gene numbers) in the European and East Asian populations, respectively (Supplementary Table 3). Only six of them were shared in different populations (Supplementary Figure 1). The *Q* − *Q* plot of the gene-based tests is shown in Supplementary Figure 2. Importantly, we identified a novel locus in the European population (*CUX2*) and two loci in the East Asian population (*CUX2* and *OAS3*) (Table 1 and Figure 1). *CUX2*, located on chromosome 12q23.13, participates in the proliferation and differentiation of higher vertebrates [35]. *CUX2* is usually expressed in the nervous system, whose disturbance is associated with the occurrence of many neurological diseases [36]. Sinner et al. have shown that *CUX2* contributes to atrial fibrillation, which confirms the association between neurological diseases and cardiovascular diseases [37]. In addition to *CUX2*, which is a shared susceptibility locus in different populations, *OAS3* shows its East Asian population specificity (Table 1). *OAS3* is one of the key antiviral factors induced by IFN, but it is also related to some characteristic factors of cardiovascular disease [38, 39]. This would help us better understand the complex relationship between CAD and other human diseases.

### 3.2. rs599839 Was a Population-Specific Variant of CAD

**Figure 2:**
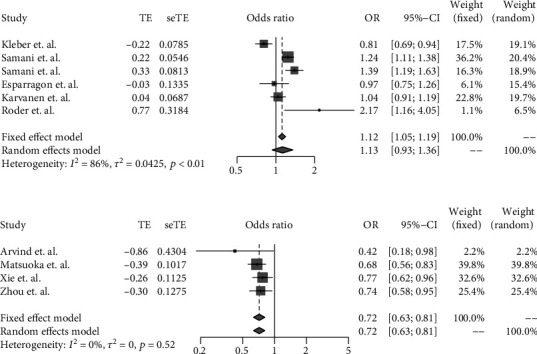
Meta-analysis of the association between rs599839 (*PSRC1*) and CAD.

We integrated the previously published GWAS and our gene-based calculation of Eurasian-specific risk loci, and rs599839 (*PSRC1*), rs17465637 (*MIA3*), rs4977574 (*CDKN2A/B*, *ANRIL*), and rs1746048 (*CXCL12*) met the meta-analysis criteria (Supplementary Table 1). We used a total of 28 independent studies from 23 published literatures, all of which tried to use additive models to evaluate the odds ratio (OR) of genetic variants on CAD to minimize heterogeneity. Of the four genetic variants, only rs599839 (*PSRC1*) showed significant variation among different populations by random-effects models. rs599839 (*PSRC1*) was a protective variant of CAD in East Asian populations (OR_ASN_ = 0.72, 95% CI: 0.63-0.81) but a risk factor for CAD in European populations (OR_EUR_ = 1.13, 95% CI: 0.93-1.36) (Figure 2). We found no unacceptable publication bias and heterogeneity through the funnel plot (Supplementary Figure 3). However, the polymorphisms of other variants are positively correlated with the risk of CAD in any population (Supplementary Figure 4, Supplementary Figure 5, and Supplementary Figure 6).

### 3.3. Cholesterol Metabolism Contributed to CAD in Both Populations

**Figure 3:**
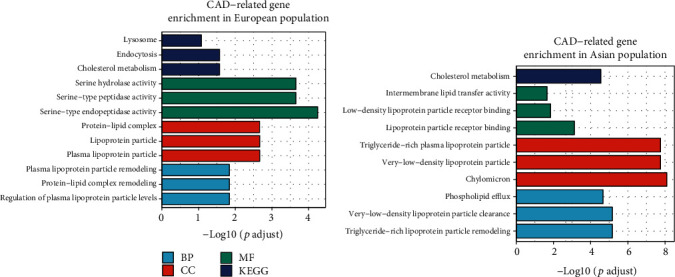
GO and KEGG enrichment of CAD-related genes.

We enriched CAD-related genes in the GO and KEGG pathway and found the evidence of differences among different populations from biological process, cellular component, and molecular function. The CAD-related genes of the European population were mainly enriched in plasma lipoprotein and serine activity pathways, while the East Asian population in triglyceride-rich lipoprotein particle pathways (Figure 3). Only cholesterol metabolism contributed to CAD in both populations (Figure 3). High total cholesterol in the blood, high low-density lipoprotein cholesterol (LDL-C), and low high-density lipoprotein cholesterol (HDL-C) were considered to be important risk factors for CAD [40]. For the tissue-specific expression analysis (TSEA), risk genes of the East Asian population were significantly expressed in cervix uteri (Supplementary Figure 7 and Supplementary Figure 8). No specific distributions were enriched in the European population, and the top three were the heart, blood vessel, and brain (Supplementary Figure 7).

### 3.4. Regulatory Element Enrichment Analysis in Noncoding Regions

**Figure 4:**
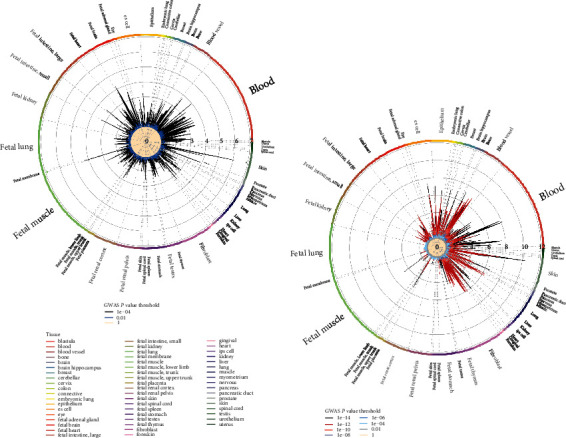
Regulatory element enrichment analysis of CAD susceptibility loci located in noncoding regions: (a) European; (b) Asian. ENCODE and Roadmap Epigenomics DNase I hypersensitive cell lines were enriched. The size of the letters outside the circle is proportional to the number of cell lines, and the different colors inside the circle are the thresholds of different *P* values of GWAS.

In order to explore how noncoding region variants regulated the occurrence of CAD in different populations, we performed the enrichment of regulatory elements in DHSs, histone modifications, and transcription factor binding sites (Figure 4) [29]. We found that CAD susceptibility sites (*P* < 0.001) were significantly enriched in DHS of blood cells (OR_EUR_ = 2.69, *P*_EUR_ = 0.036; OR_ASN_ = 1.38, *P*_ASN_ = 4.5*E* − 04) and blood vessels (OR_EUR_ = 3.05, *P*_EUR_ = 0.016; OR_ASN_ = 1.40, *P*_ASN_ = 4.8E − 04) in different lineages (Figure 3). Surprisingly, DHSs in skin tissues also seemed to play an important role in the pathogenesis of CAD (OR_EUR_ = 6.05, *P*_EUR_ = 8.0E − 05; OR_ASN_ = 1.34, *P*_ASN_ = 4.3*E* − 04). It was a novel view building a connection between skin and CAD.

Besides DHSs, blood was also rich in transcription factor binding sites (Supplementary Figure 9). However, other regulatory elements had significant population-specific differences. Genetic annotations showed that CAD variants in the European population were mainly enriched in 3′UTR and 5′UTR, while those in the East Asian population were significantly enriched in exons (Supplementary Figure 10). Generally, the significance of enrichment (*P* value) in the East Asian population was higher than that in the European population (Supplementary Figure 11). More details were presented in the Supplementary Table 4-5.

### 3.5. Population-Specific Differences of Causal Genes

**Figure 5:**
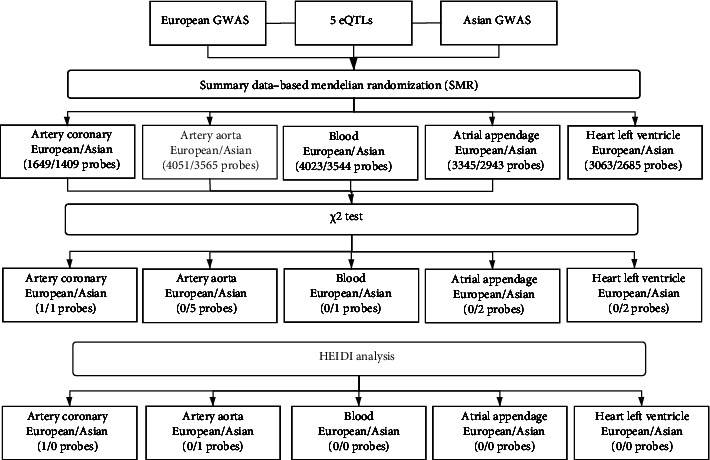
The experimental process of SMR. SMR analyses were performed between 5 eQTLs and GWAS from European and Asian populations, respectively. The probes were screened by significance test and HEIDI analysis.

We selected five related eQTLs, including artery coronary (22950 probes), artery aorta (22366 probes), blood (19432 probes), heart atrial appendage (21733 probes), and heart left ventricle (20155 probes) tissues, for SMR analysis in the European and East Asian populations. We mapped all the susceptibility loci to eQTL target genes in cis-regions and then linked them to CAD. Manhattan plots of SMR tests for association between gene expression and CAD could be found in Supplementary Figure Figure 12. In order to test the significance of each probe, the FDR threshold was set to 0.05/probe numbers. In the above 10 studies, only *NBEAL1* (*P*_SMR_ = 8.42*E* − 06, *P*_HEIDI_ = 0.53) in the European population and *FGD6* (*P*_SMR_ = 5.70*E* − 06, *P*_HEIDI_ = 0.20) in the Asian population passed the threshold of the *χ*^2^ test and HEIDI test (Figure 5 and Table 2). In other words, *NBEAL1* and *FGD6* were population-specific pleiotropic/causal genes of CAD (regional plots for them are shown in Figure 1). In addition, *PHACTR1*, *ADAMTS7*, *RPH3A*, *ABO*, *RP11-378J18.8*, and *RP1-257A7.5* were linkage genes (two distinct causal variants in top-associated cis-eQTL concurrently, one affecting gene expression and the other affecting trait variation).

*NBEAL1* was known as a susceptibility locus of CAD, which was highly expressed in the heart and artery [41]. It affected cholesterol metabolism and LDL uptake by regulating the activity of *SREBP2* in cells and then affected the pathogenesis of CAD afterwards [42]. However, *FGD6* might be a novel CAD susceptibility locus, which regulated the proangiogenic activity in vitro. For *NBEAL1* and *FGD6*, the significant population-specific genes, we further analyzed their regulation mechanism on CAD. Functionally relevant genes and regulatory genes adjacent to it were pinpointed on the chromosome (Supplementary Figure 13). The effect sizes of SNPs showed that the overexpression of *NBEAL1* was associated with the increased risk of CAD, while overexpression of *FGD6* was associated with decreased CAD level (Supplementary Figure 14). It was worth noting that the top SNP of *NBEAL1* (rs2351524) failed to pass the genome-wide association level (*P* < 5*E* − 08). Thus, we lent support to the theory that genetic variants and genes with low associations with traits might affect traits as well.

## 4. Discussion

In this study, the population-specific differences of CAD were revealed from various aspects, including susceptibility loci, risk genetic variants, biological pathways, and regulatory elements in the noncoding region. The differences in CAD between the European population and the East Asian population were obvious from every aspect. We provided new insights from the perspective of coding and noncoding regions at the same time, which were more novel than previous studies.

Sex and ancestry were the potential causes of CAD differences [18, 43–45]. However, previous studies were limited to small sample sizes, which made it difficult to overcome the defects of heterogeneity. Therefore, our study had many advantages. First of all, we selected two summary-level data from independent GWAS, both of which were each composed of the same ancestry. Mixing different lineages together for meta-analysis might blur the differences among different populations. Secondly, we used a gene-based test to obtain the risk loci effectively that were often overlooked in GWAS (*P* > 5*E* − 08) and identified two novel susceptibility loci for CAD (*CUX2*, *OAS3*). Finally, we fully revealed the population-specific differences of CAD from various perspectives.

However, our study also had certain limitations. The lack of large-scale GWAS in East Asia led to only the Japanese ancestry being used to replace the East Asian ancestry. In addition, the donors of the GTEx project were of multiple descents including European (85.3%), African (12.3%), and Asian (1.4%) [21]. We expect more non-European eQTL studies to complement our deficiencies. Secondly, little evidence showed that the two novel susceptibility loci (*CUX2* and *OAS3*) contributed to CAD in previous studies, so further biological experiment verification was necessary. Finally, SMR analysis had difficulty in distinguishing causal genes from pleiotropic genes. *NBEAL1* and *FGD6*, the population-specific pleiotropic/causal genes for CAD, needed further exploration.

## 5. Conclusions

In this study, we integrated multiple-omics data to mine the population-specific differences for CAD. We provided a new insight into the genetic mechanism of nonwhite and genetic evidence for CAD precision medicine in different populations. We call for more large-scale GWAS research on CAD with different lineages to achieve accurate treatment of specific gene targets, especially those of non-European lineages that have been previously neglected.

## Figures and Tables

**Table 1:** New loci identified by gene-based test.

Population	Chr	Gene	nSNPs	Test	Top-SNP	Ref	Alt	EAF	Beta	*P* value
European	12	CUX2	113	1051.29	rs4766453	C	T	0.74	0.080	1.80*E*-06
East Asian	12	CUX2	360	6077.64	rs79105258	A	C	0.75	0.269	6.55*E*-32
	12	OAS3	336	4020.37	rs3937435	A	G	0.65	-0.138	2.04*E*-12

**Table 2:** Genes that are causally associated with the risk of CAD.

Population	eQTL	Probe ID	CHR	Gene	Top SNP	p_GWAS	p_eQTL	b_SMR	p_SMR	p_HEIDI
European	Artery coronary	ENSG00000144426.14	2	***NBEAL1***	rs2351524	0.4860822	2.77*E*-09	-0.314	8.42*E*-06	0.533
East Asian	Artery aorta	ENSG00000272750.1	1	*RP11-378J18.8*	rs2291832	3.672*E*-10	2.01*E*-30	-0.161	3.79*E*-08	4.54*E*-04
		ENSG00000112137.12	6	*PHACTR1*	rs9349379	1.177*E*-13	1.68*E*-11	-0.616	6.12*E*-07	1.72*E*-07
		ENSG00000272379.1	6	*RP1-257A7.5*	rs9349379	1.177*E*-13	4.01*E*-10	-0.576	1.72*E*-06	NA
		ENSG00000180263.9	12	***FGD6***	rs7954260	8.589*E*-08	1.49*E*-17	0.228	5.70*E*-06	0.198
		ENSG00000136378.10	15	*ADAMTS7*	rs7173743	2.741*E*-08	4.94*E*-13	-0.329314	1.06*E*-05	0.00590
	Artery coronary	ENSG00000272750.1	1	*RP11-378J18.8*	rs17163358	1.854*E*-09	2.78*E*-10	-0.202	1.32*E*-05	0.00121
	Blood	ENSG00000089169.10	12	*RPH3A*	rs7979186	5.75*E*-09	7.75*E*-55	-0.150	4.95*E*-08	6.11*E*-04
	Heart atrial appendage	ENSG00000272750.1	1	*RP11-378J18.8*	rs61824331	3.534*E*-08	3.07*E*-20	-0.172	2.20*E*-06	0.0129
		ENSG00000175164.9	9	*ABO*	rs643434	5.315*E*-08	5.00*E*-22	0.170	2.20*E*-06	2.07*E*-05
	Heart left ventricle	ENSG00000272750.1	1	*RP11-378J18.8*	rs2291832	3.672*E*-10	1.41*E*-23	-0.155	1.08*E*-07	3.71*E*-04
		ENSG00000175164.9	9	*ABO*	rs657152	6.34*E*-09	6.84*E*-20	0.214	9.14*E*-07	1.053*E*-05

## Data Availability

All data generated or analyzed during this study are included in this article.

## Supplementary Materials

Supplementary Materials Supplementary Figure 1: Venn diagram of the number of risk genes in the European population and East Asian population. Supplementary Figure 2: *Q* − *Q* plot of the gene-based test computed by MAGMA. Supplementary Figure 3: funnel plot of meta-analysis of rs599839 (PSRC1). Supplementary Figure 4: meta-analysis of the association between rs17465637 (MIA3) and CAD. Supplementary Figure 5: meta-analysis of the association between rs4977574 (CDKN2A/B, ANRIL) and CAD. Supplementary Figure 6: meta-analysis of the association between rs1746048 (CXCL12) and CAD. Supplementary Figure 7: relationship between highly expressed genes in a specific tissue and genetic associations. Supplementary Figure 8: gene expression heat map by hierarchical clustering. Supplementary Figure 9: transcription factor binding site enrichment analysis of CAD susceptibility loci. Supplementary Figure 10: genetic annotation analysis of CAD susceptibility loci. Supplementary Figure 11: histone modification enrichment analysis of CAD susceptibility loci. Supplementary Figure 12: Manhattan plots of SMR tests for association between gene expression and CAD. Supplementary Figure 13: prioritizing genes at GWAS loci using SMR analysis. Supplementary Figure 14: effect sizes of SNPs from GWAS plotted against those for SNPs from eQTL studies. Supplementary Table 1: integrating the population-specific genetic variants reported before and identified by a gene-based test in the present study. Supplementary Table 2: meta-analysis of population-specific genetic variants. Supplementary Table 3: identification of CAD risk genes by using VEGAS. Supplementary Table 4: the results of regulatory element enrichment analysis for CAD in the European population. Supplementary Table 5: the results of regulatory element enrichment analysis for CAD in the Asian population.
