# Supplementary material for: Integration of Multiple-Omics Data to Analyze the Population-Specific Differences for Coronary Artery Disease
Source: Comput Math Methods Med. 2021 Aug 17;2021:7036592. doi: 10.1155/2021/7036592 (PMC8384508; doi:10.1155/2021/7036592)

**Supplementary Figure 1. Venn diagram of the number of risk genes in European population and East Asian population.**


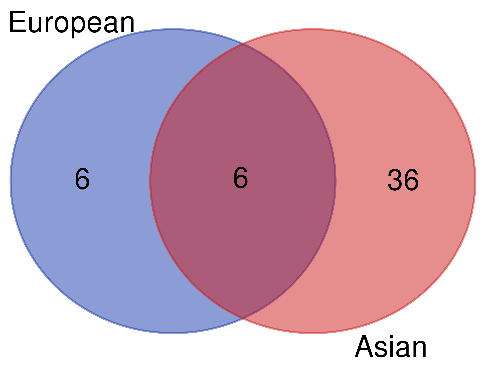


**Supplementary Figure** **2. Q-Q plot of the gene-based test computed by MAGMA.**

**A. CAD in Asian population**


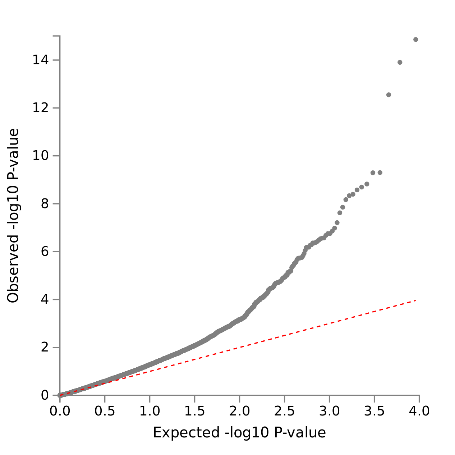


**B. CAD in European population**


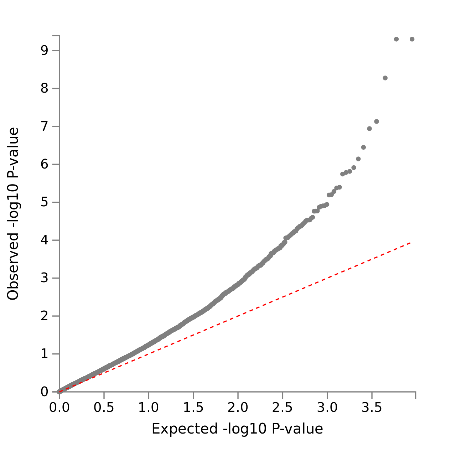


**Supplementary Figure 3. Funnel plot of meta-analysis of rs599839 (PSRC1).**

**A: European population**


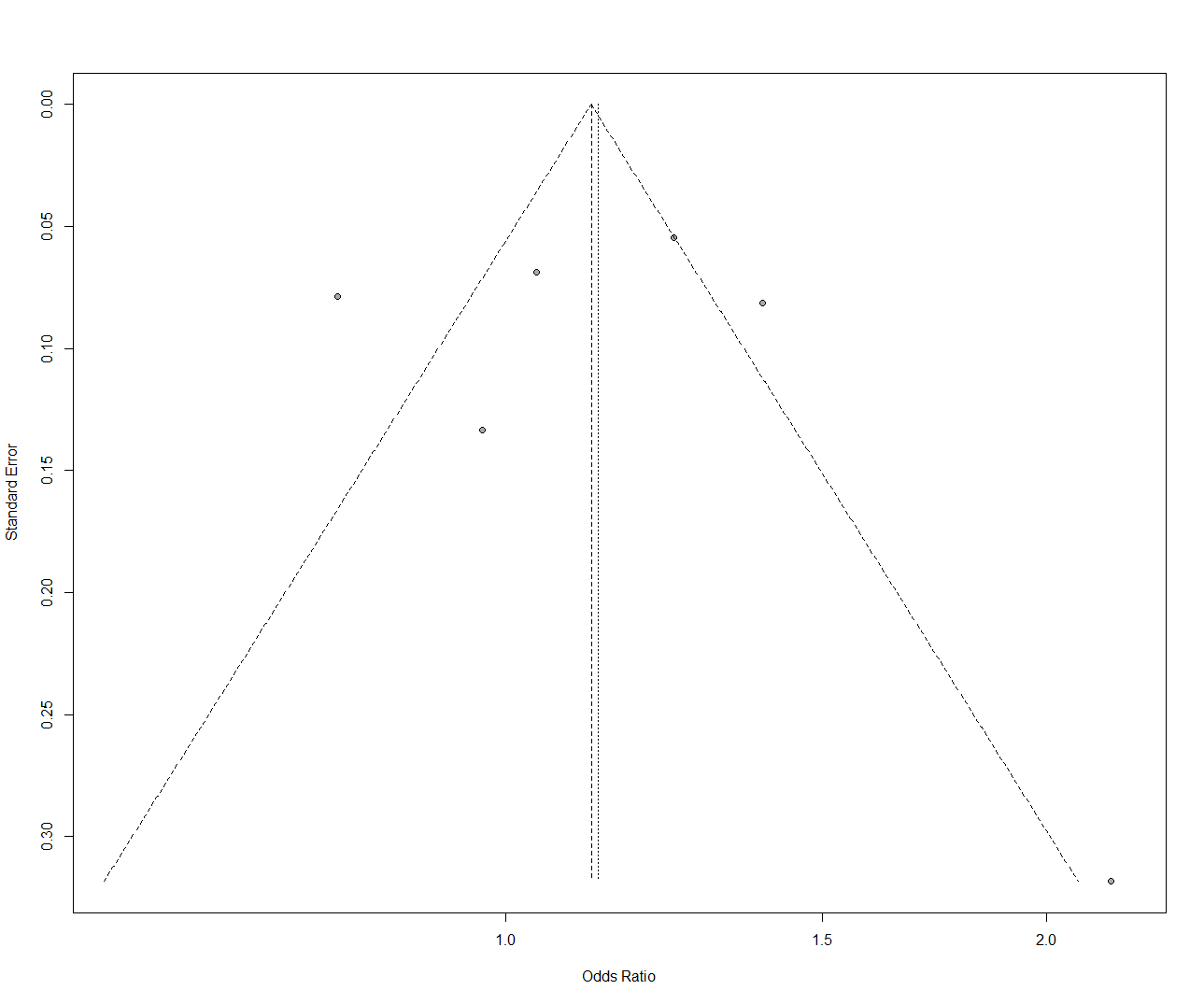


**B: Asian population**


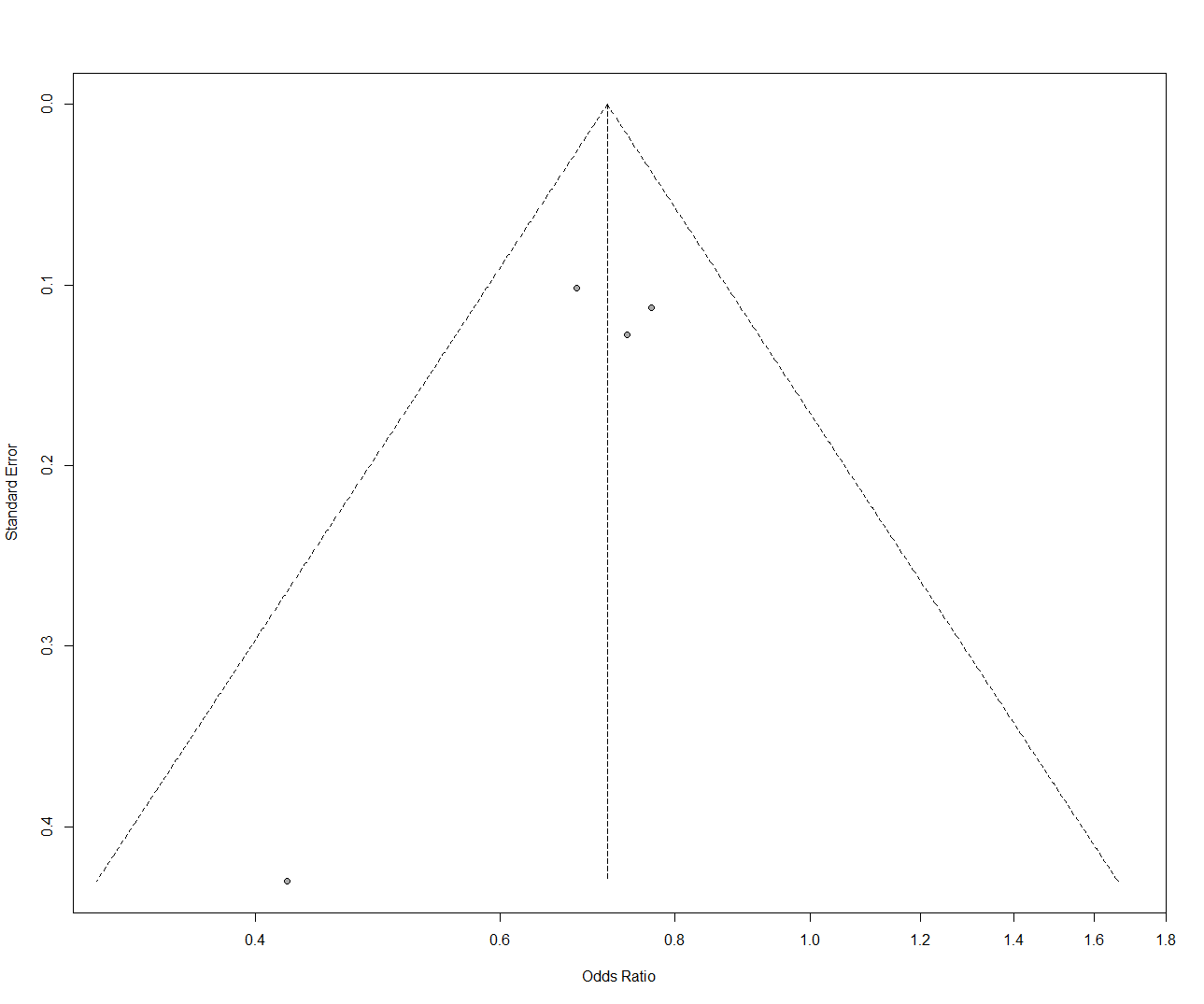


**Supplementary Figure 4. Meta-analysis of the association between rs17465637 (*MIA3*) and CAD.**

**A: European population**


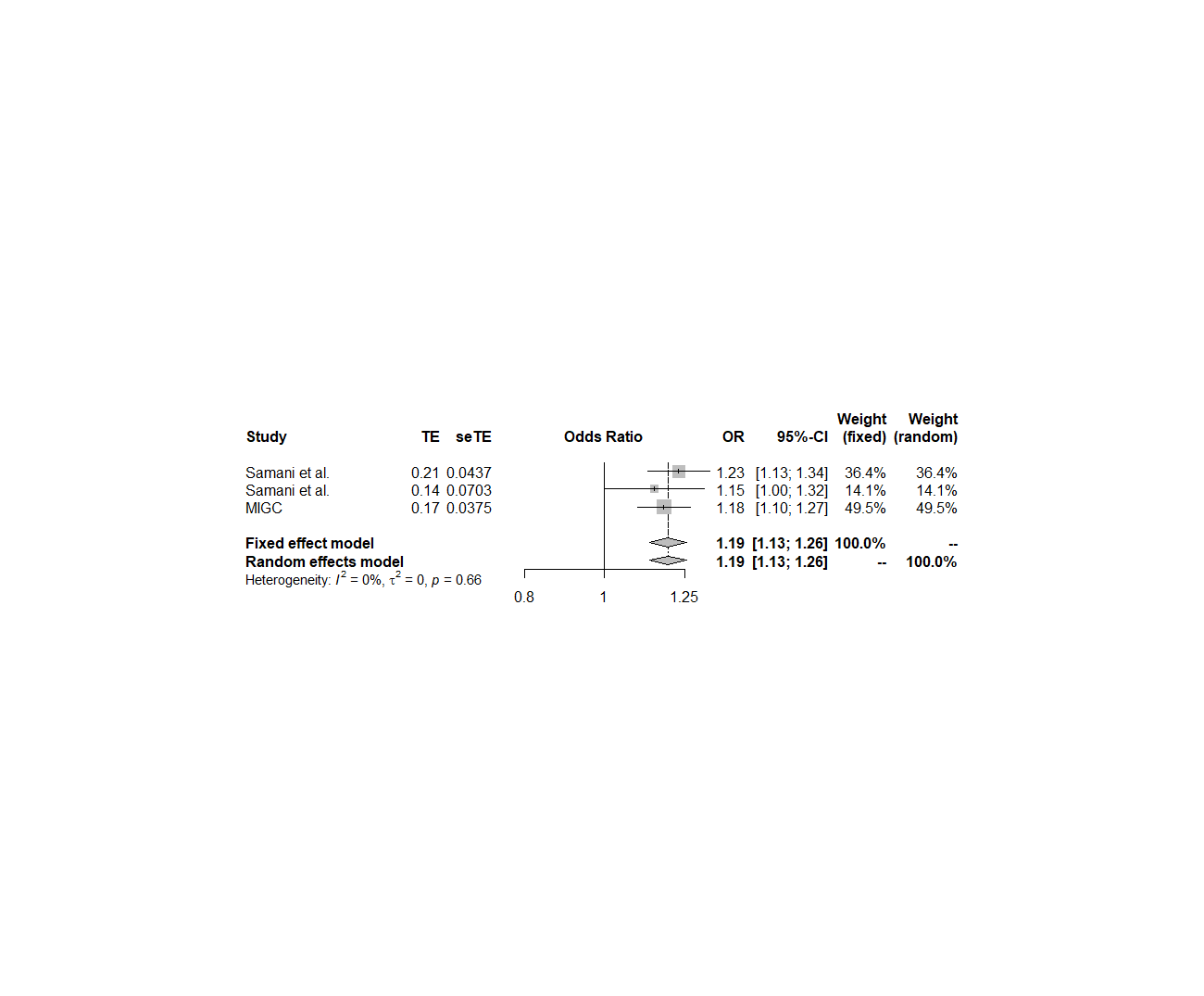


**B: Asian population**


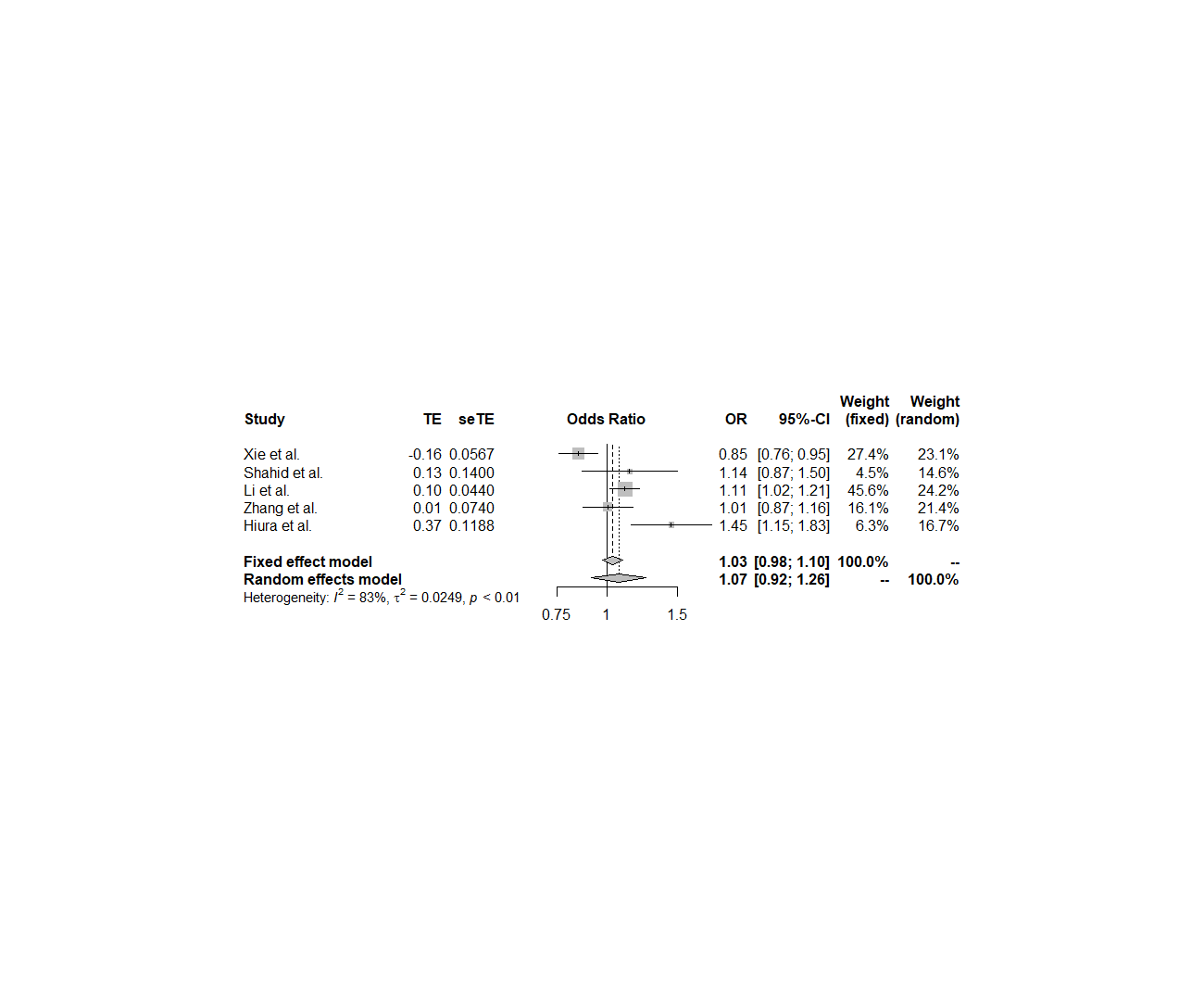


**Supplementary Figure 5. Meta-analysis of the association between rs4977574 (*CDKN2A/B*, *ANRIL*) and CAD.**

**A: European population**


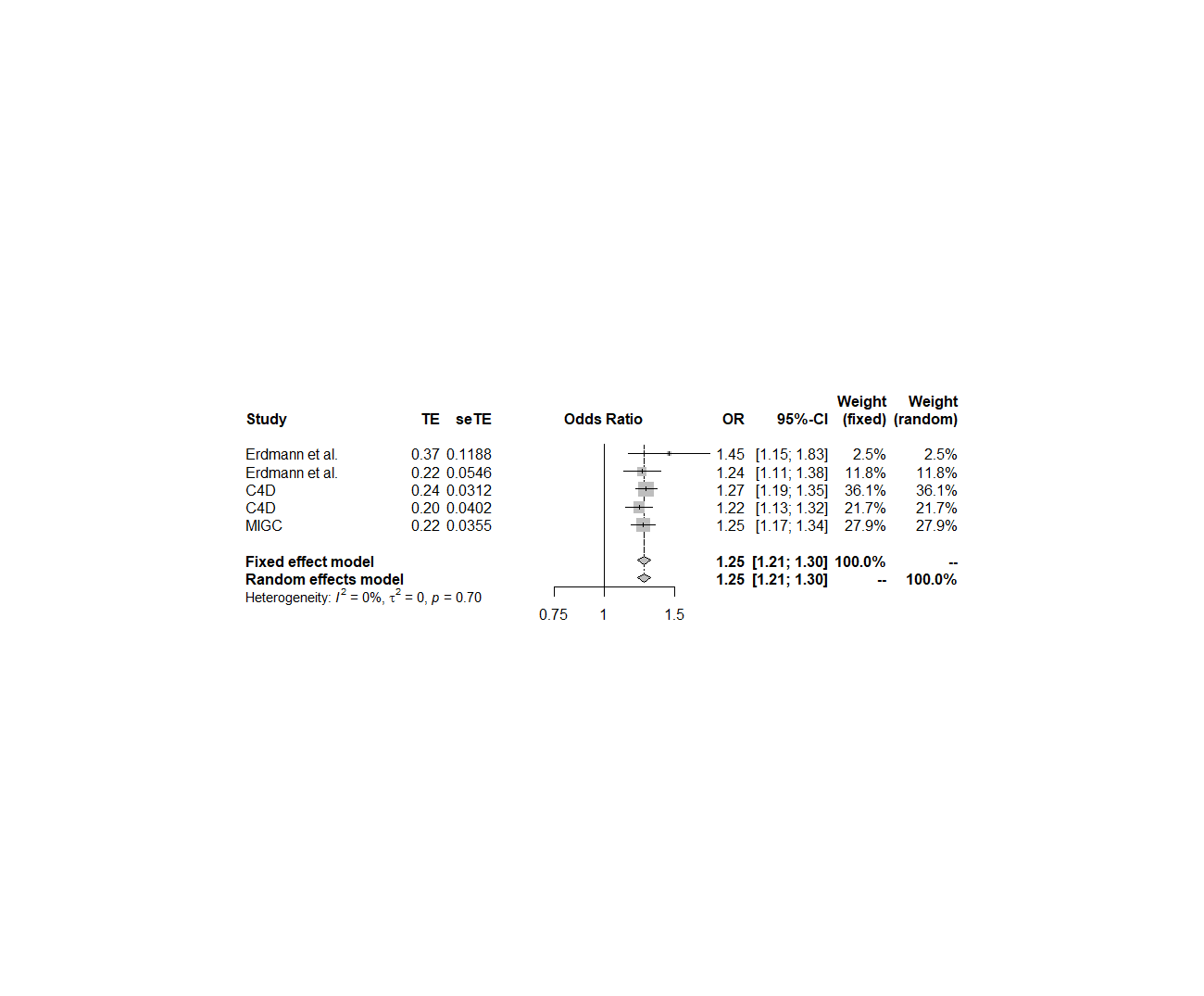


**B: Asian population**


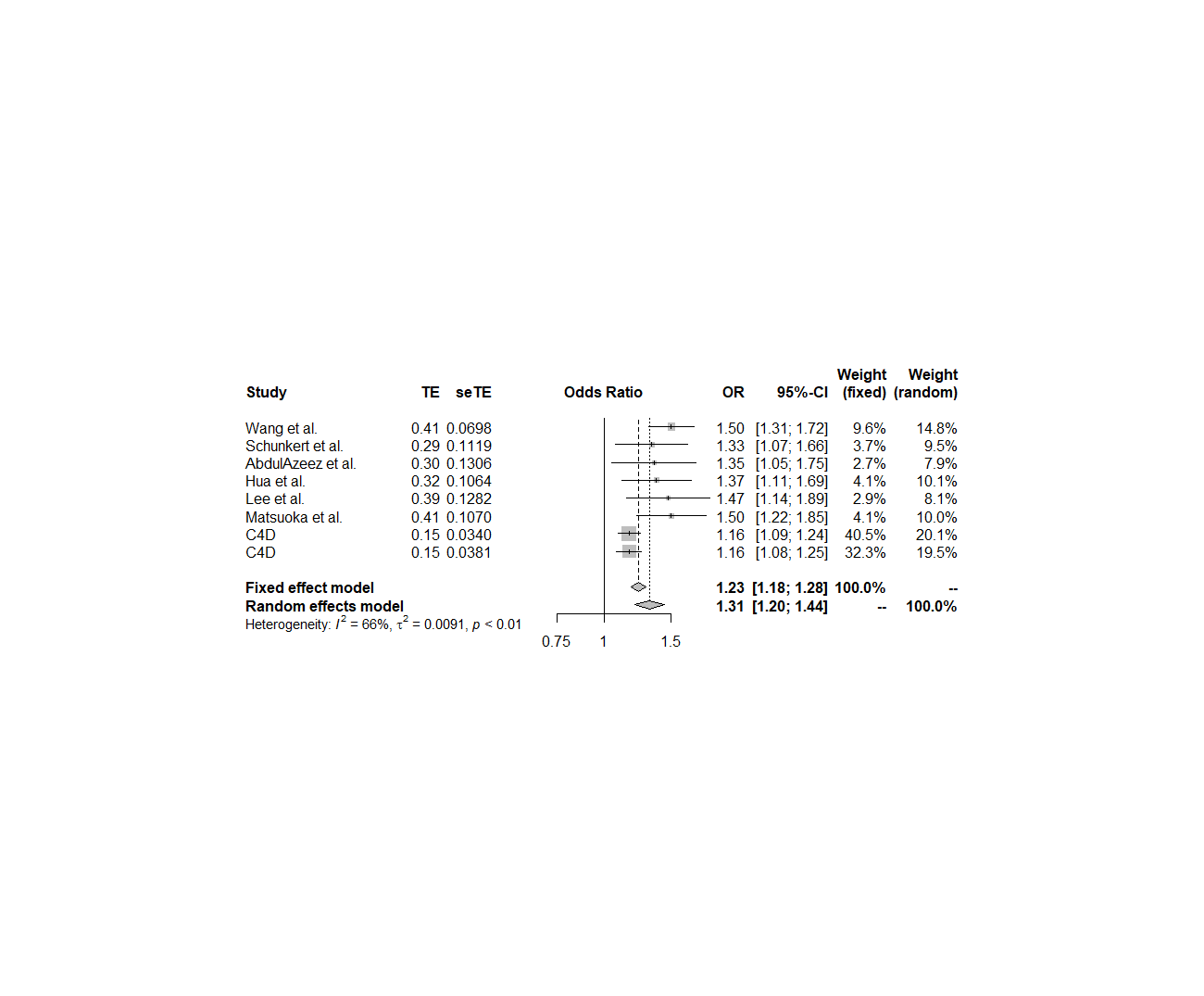


**Supplementary Figure 6. Meta-analysis of the association between rs1746048 (*CXCL12*) and CAD.**

**A: European population**


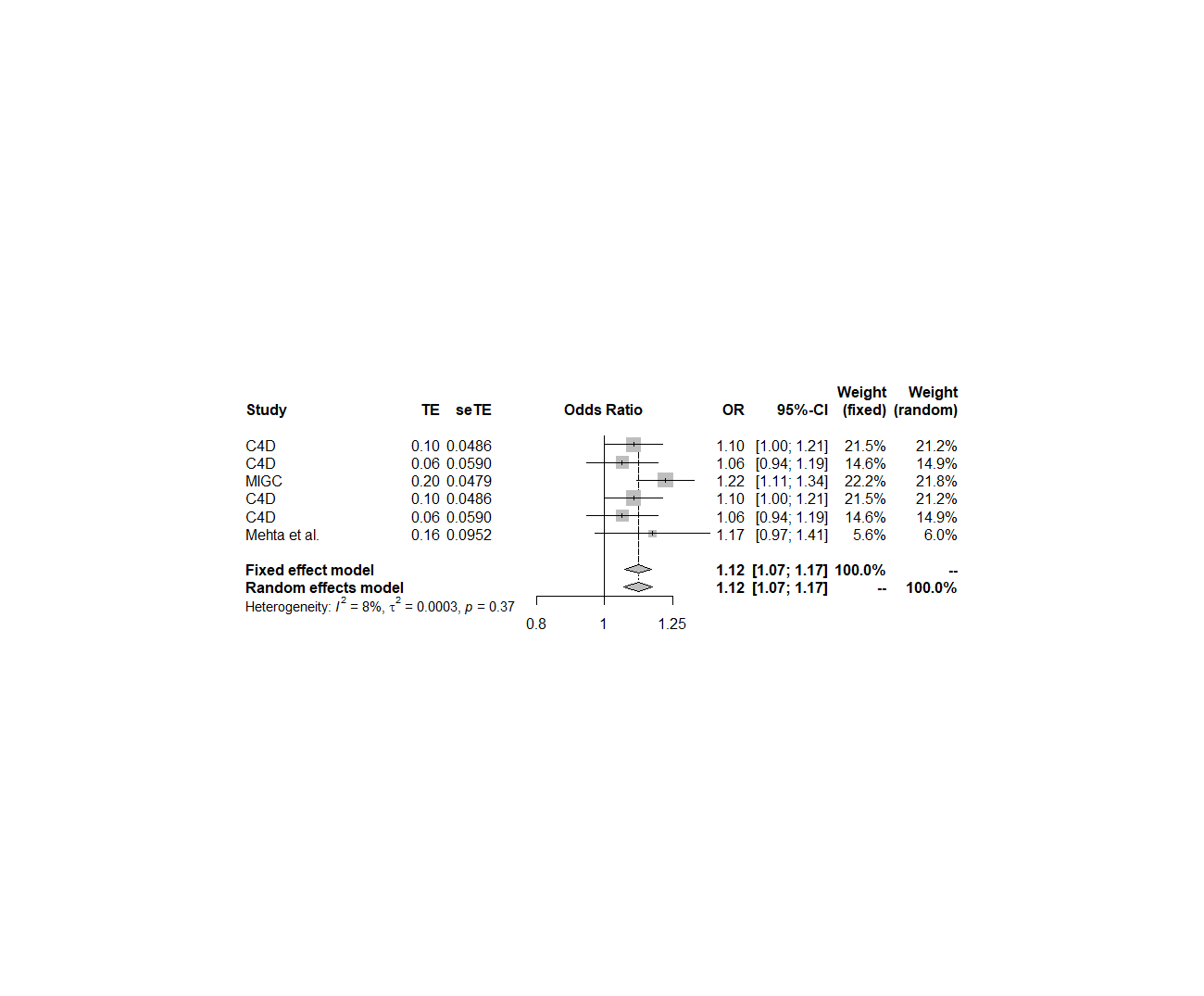


**B: Asian population**


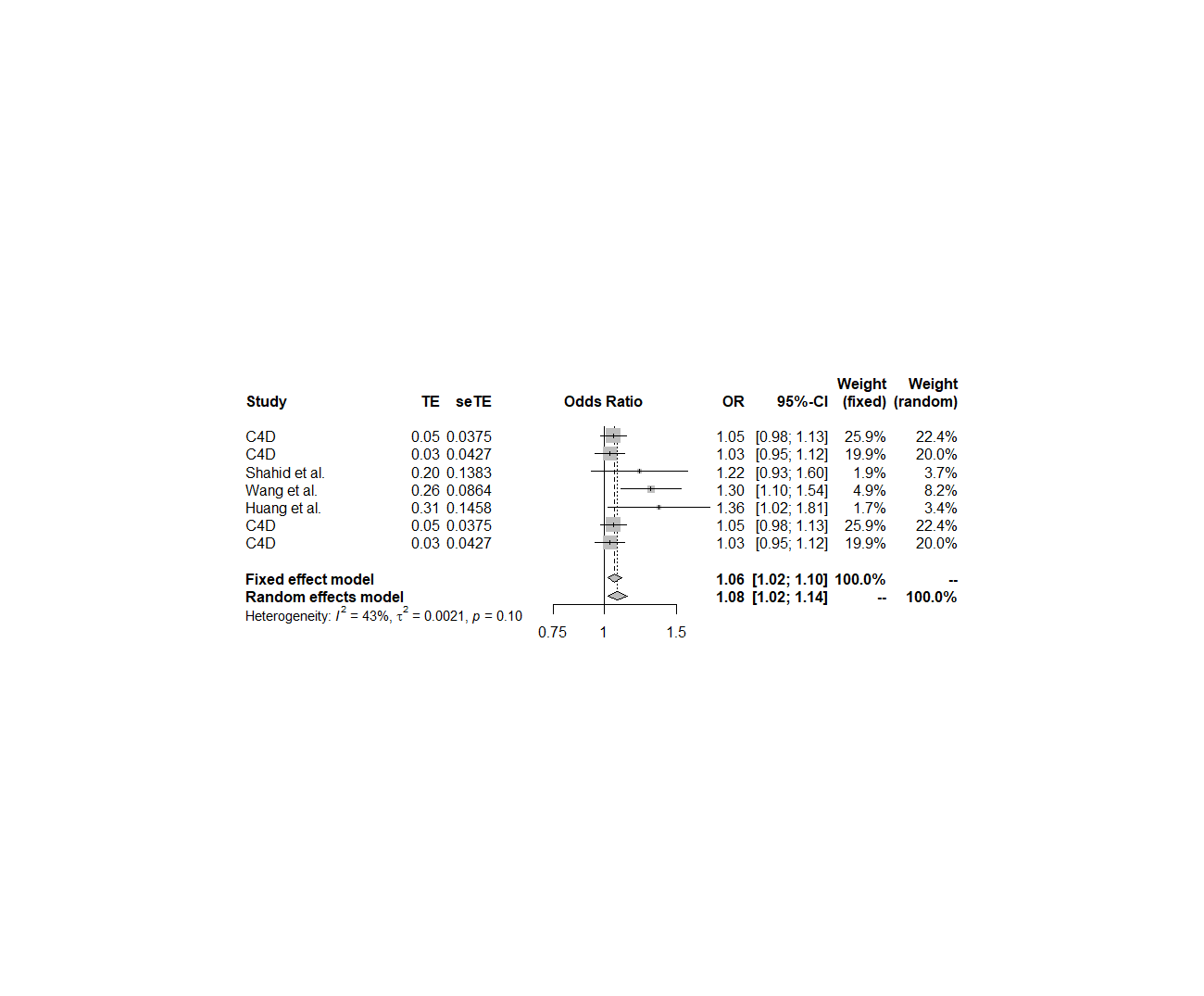


**Supplementary Figure 7. Relationship between highly expressed genes in a specific tissue and genetic associations.**

1. **European population. (B) Asian population.**


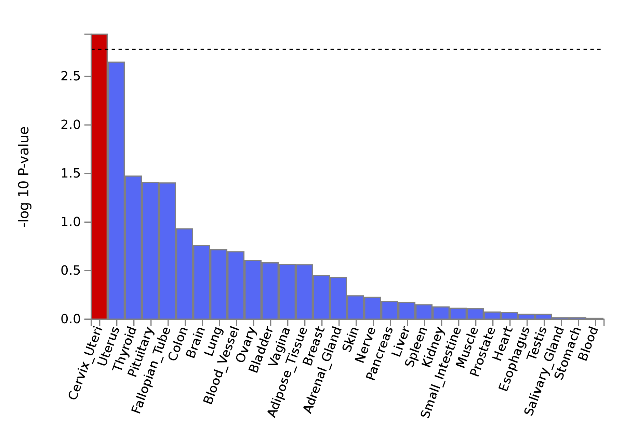


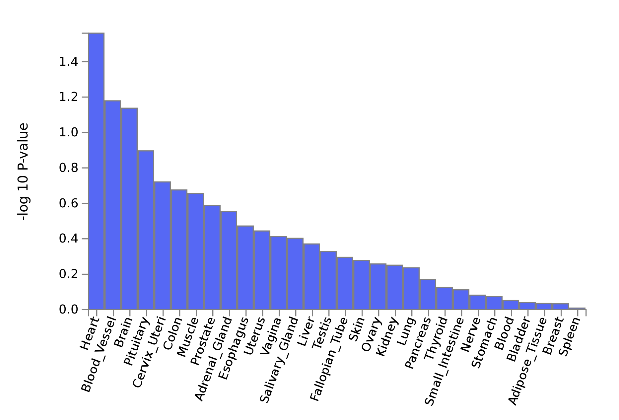


**Supplementary Figure 8. Gene expression heatmap by hierarchical clustering.**


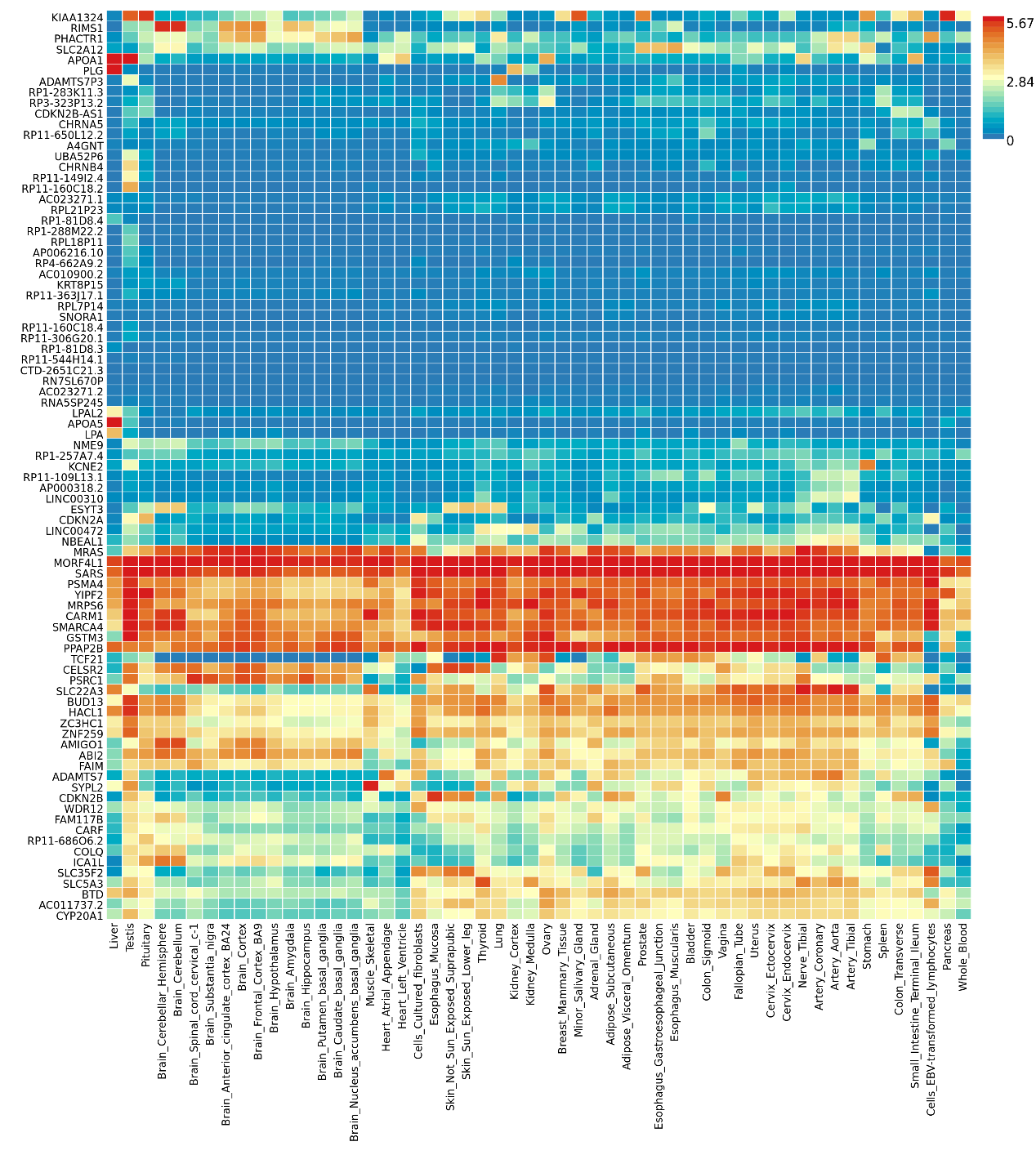


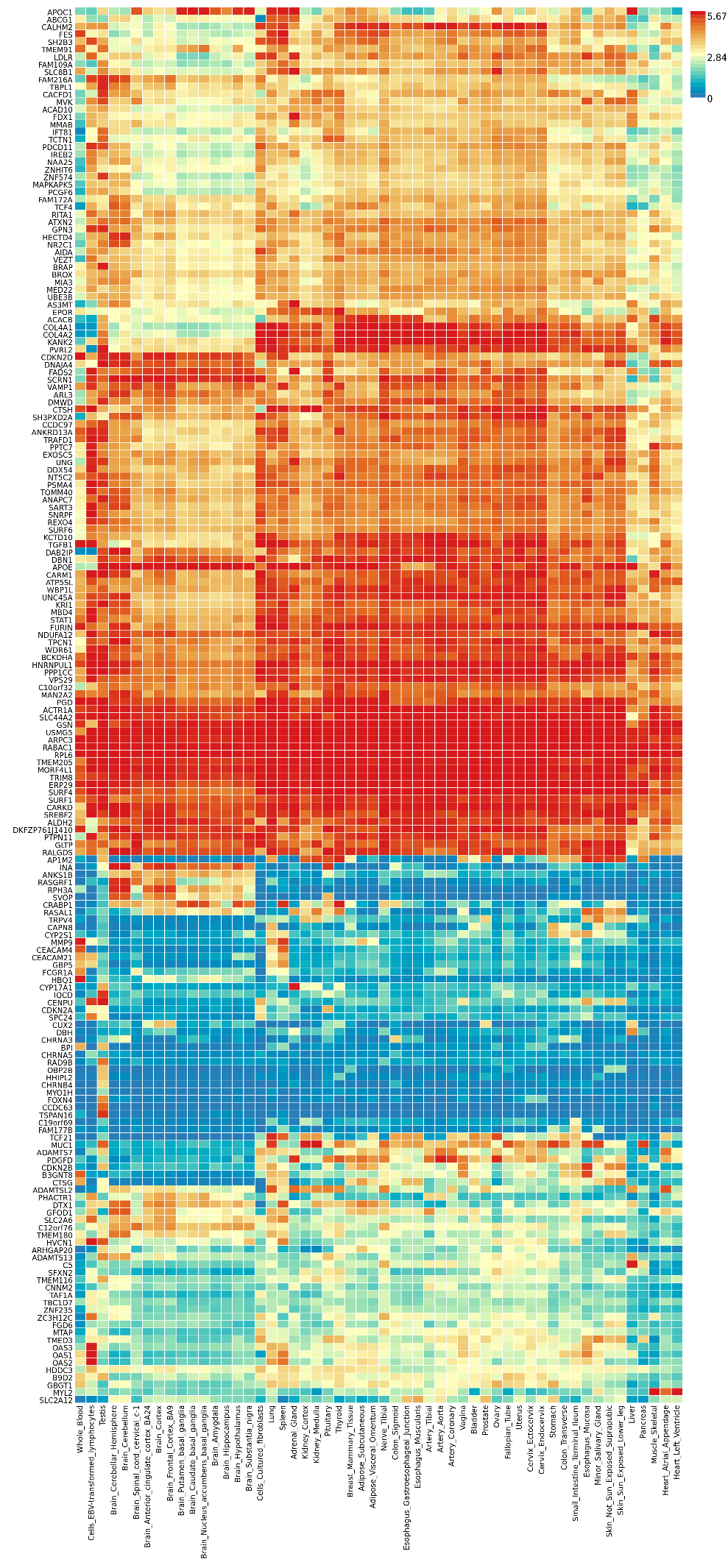


**Supplementary Figure 9. Transcription factor binding site enrichment analysis of CAD susceptibility loci. (A) European. (B) Asian.**


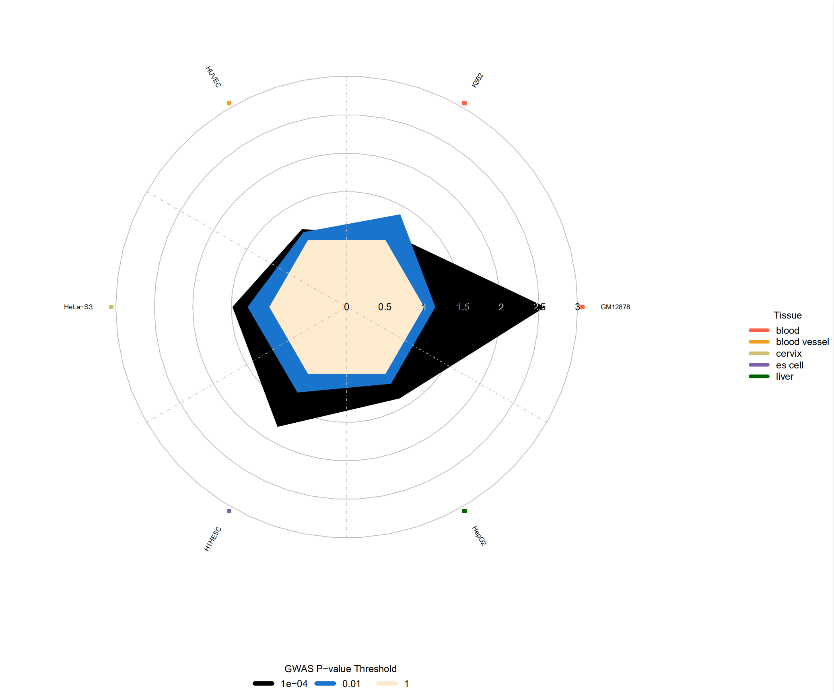


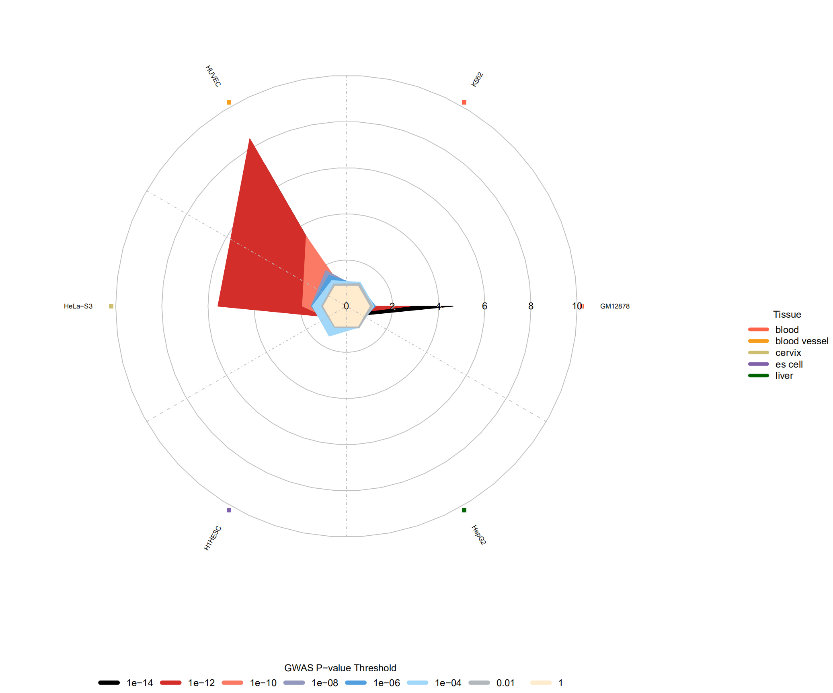


**Supplementary Figure 10. Genetic annotation analysis of CAD susceptibility loci. (A) European. (B) Asian.**


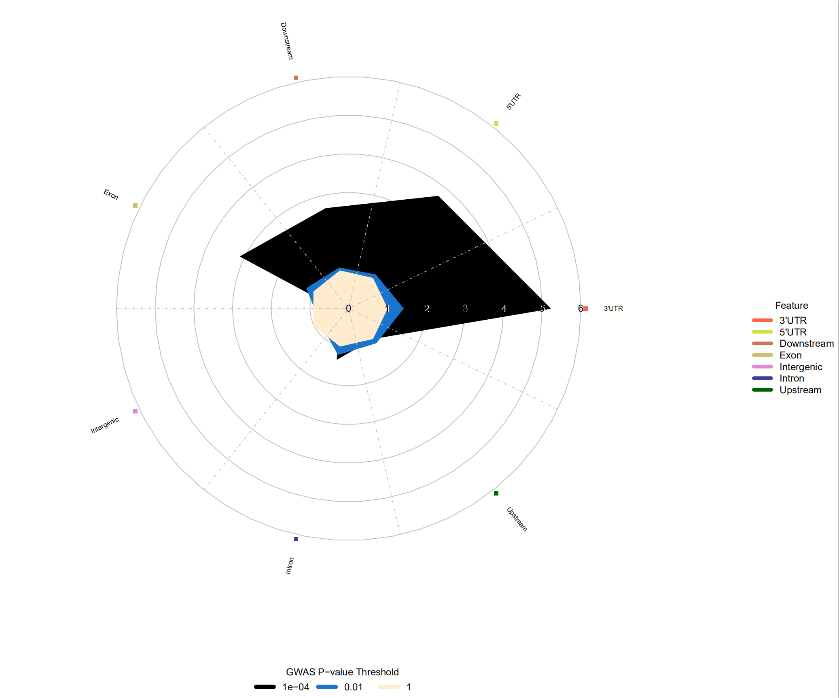


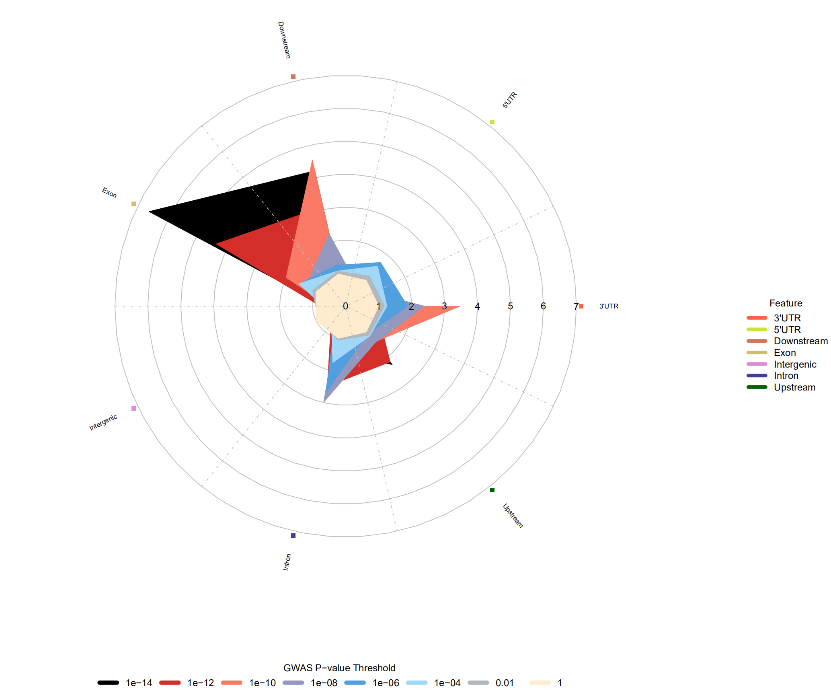


**Supplementary Figure 11.** **Histone modification** **enrichment analysis of CAD susceptibility loci. (A) European. (B) Asian.**


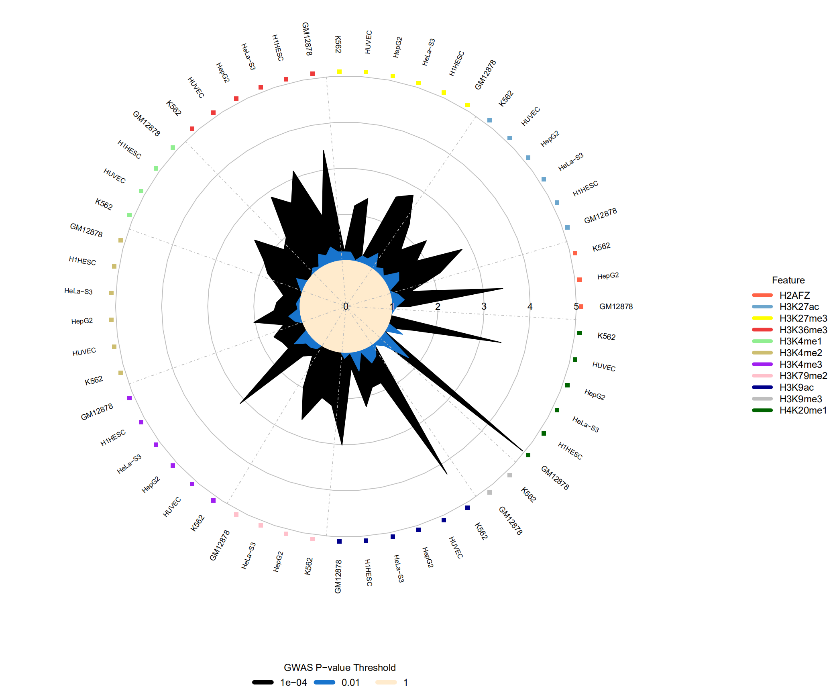


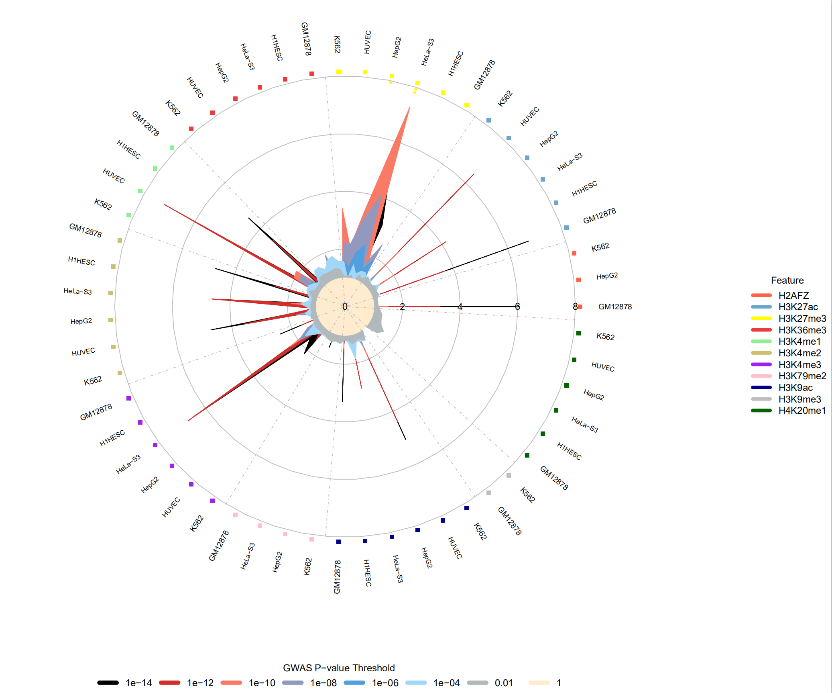


**Supplementary Figure 12.** **Manhattan plots of SMR tests for association between gene expression and CAD. Herein, we only show manhattan plots which exist pleiotropic/causal genes passed SMR test. Shown on each *y* axis are the −log_10_ *P* from SMR tests. The red horizontal lines represent the genome-wide significance level (*P_SMR_*). (A) Artery coronary in European population. (B)Artery aorta in Asian population.**


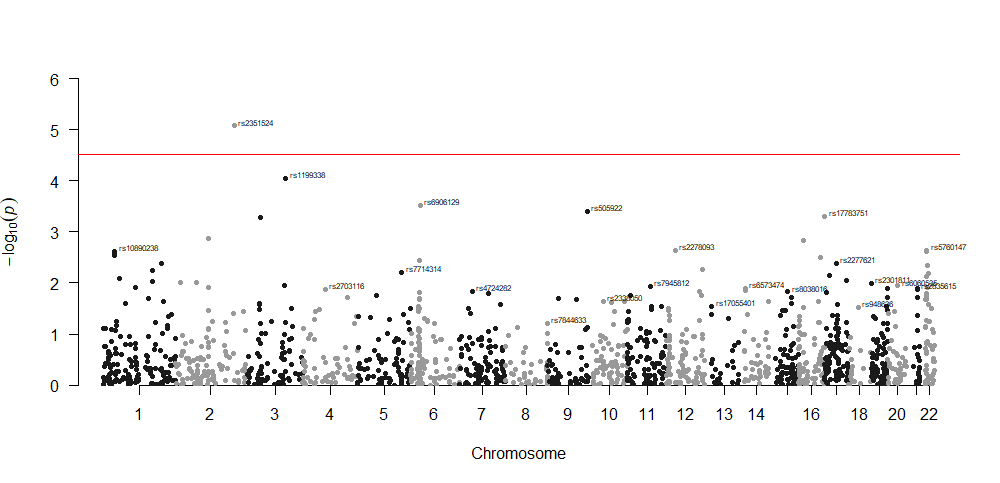


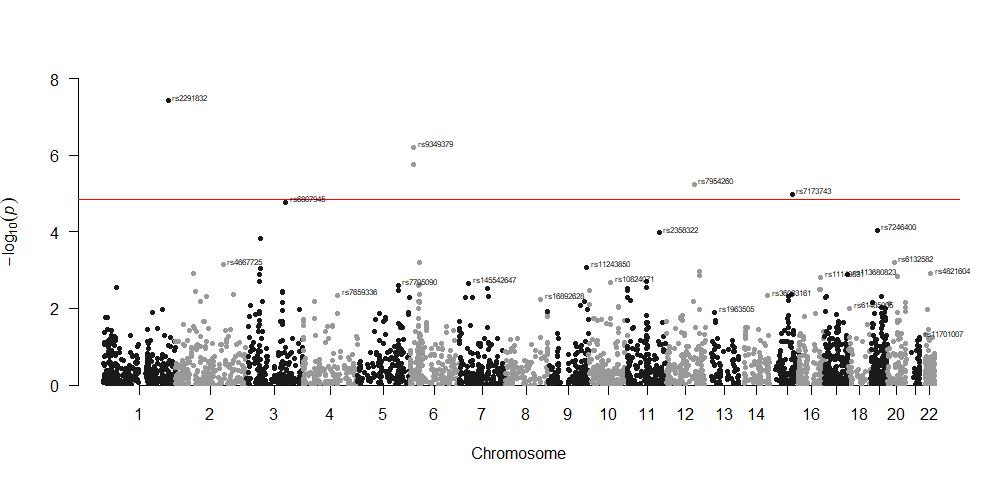


**Supplementary Figure 13. Prioritizing genes at GWAS loci using SMR analysis. (A) *NBEAL1*. (B) *FGD6*.** Top plot, brown dots represent the P-values for SNPs from the latest GWAS meta-analysis, diamonds represent the P-values for probes from the SMR test and triangles represent probes without a cis-eQTL at *P_eQTL_* < 5e-08. Highlighted in red is the gene (*NBEAL1* and *FGD6*) that passed the SMR and HEIDI tests.


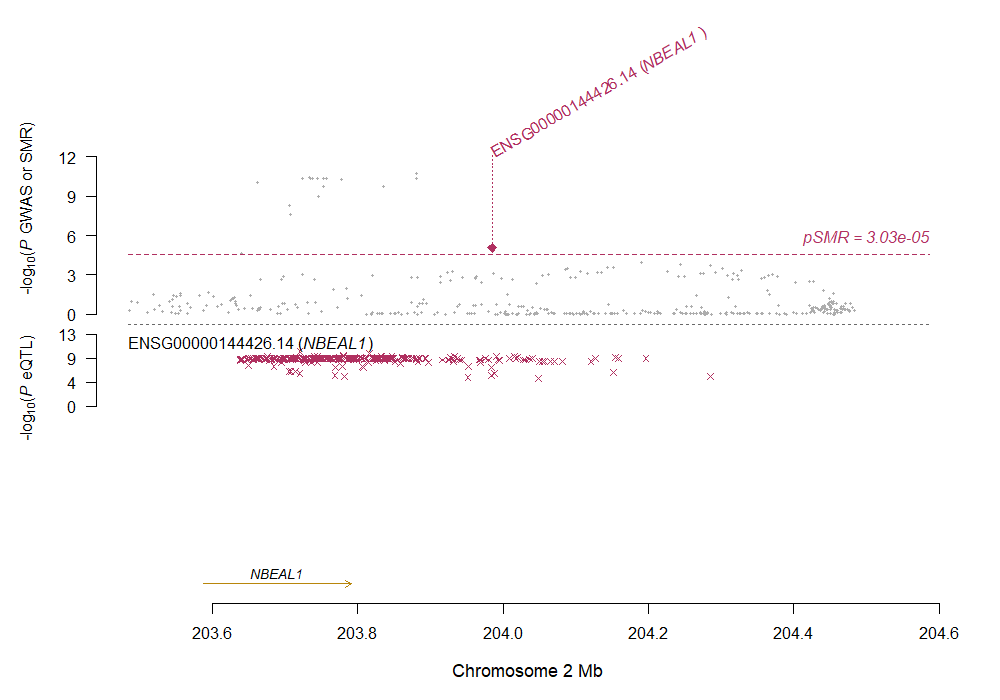


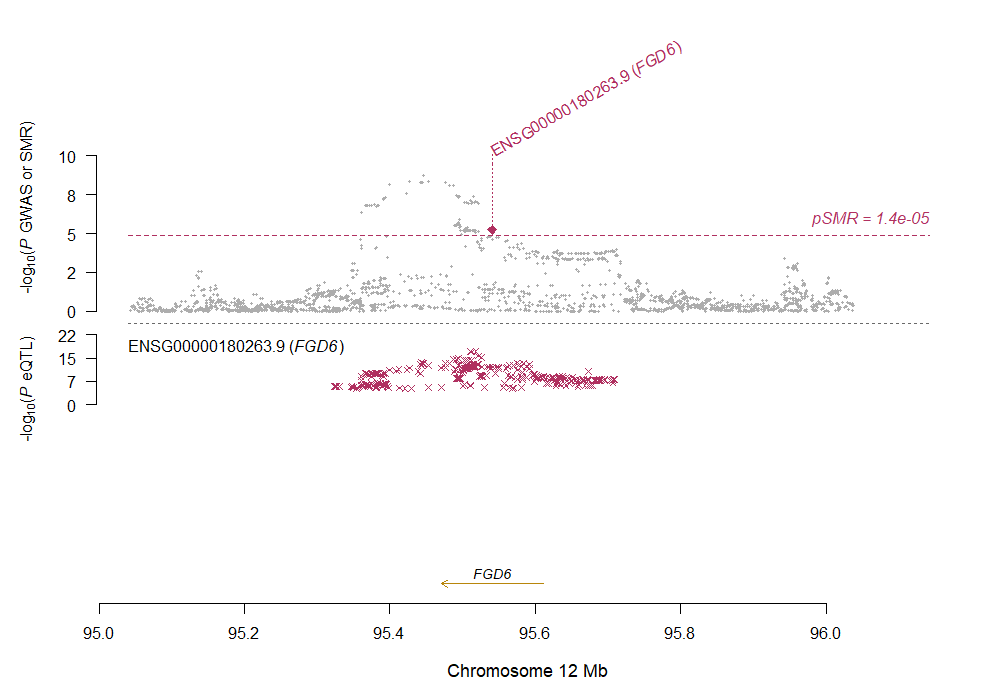


**Supplementary Figure 14. Effect sizes of SNPs from GWAS plotted against those for SNPs from eQTL studies. (A) *NBEAL1*. (B) *FGD6*.** The orange dashed lines represent the estimate of *b_xy_* at the top cis-eQTL. Error bars are the standard errors of SNP effects.


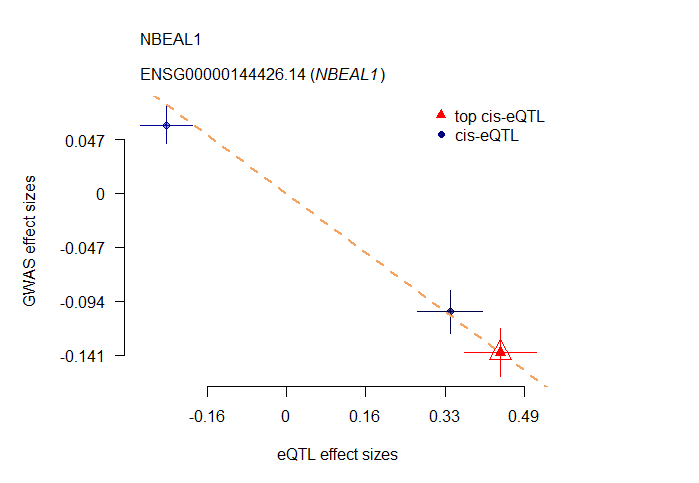

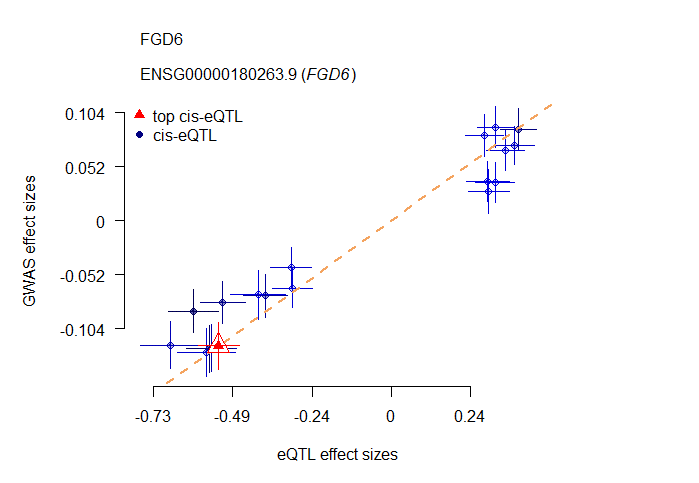

Supplement: Supplementary Materials — Supplementary Figure 1: Venn diagram of the number of risk genes in the European population and East Asian population. Supplementary Figure 2: Q − Q plot of the gene-based test computed by MAGMA. Supplementary Figure 3: funnel plot of meta-analysis of rs599839 (PSRC1). Supplementary Figure 4: meta-analysis of the association between rs17465637 (MIA3) and CAD. Supplementary Figure 5: meta-analysis of the association between rs4977574 (CDKN2A/B, ANRIL) and CAD. Supplementary Figure 6: meta-analysis of the association between rs1746048 (CXCL12) and CAD. Supplementary Figure 7: relationship between highly expressed genes in a specific tissue and genetic associations. Supplementary Figure 8: gene expression heat map by hierarchical clustering. Supplementary Figure 9: transcription factor binding site enrichment analysis of CAD susceptibility loci. Supplementary Figure 10: genetic annotation analysis of CAD susceptibility loci. Supplementary Figure 11: histone modification enrichment analysis of CAD susceptibility loci. Supplementary Figure 12: Manhattan plots of SMR tests for association between gene expression and CAD. Supplementary Figure 13: prioritizing genes at GWAS loci using SMR analysis. Supplementary Figure 14: effect sizes of SNPs from GWAS plotted against those for SNPs from eQTL studies. Supplementary Table 1: integrating the population-specific genetic variants reported before and identified by a gene-based test in the present study. Supplementary Table 2: meta-analysis of population-specific genetic variants. Supplementary Table 3: identification of CAD risk genes by using VEGAS. Supplementary Table 4: the results of regulatory element enrichment analysis for CAD in the European population. Supplementary Table 5: the results of regulatory element enrichment analysis for CAD in the Asian population. [file 7036592.f1.zip › CAD_Supplementary_Figure.docx]
