# Supplementary material for: Integration of Multiple-Omics Data to Analyze the Population-Specific Differences for Coronary Artery Disease
Source: Comput Math Methods Med. 2021 Aug 17;2021:7036592. doi: 10.1155/2021/7036592 (PMC8384508; doi:10.1155/2021/7036592)
Supplement: Supplementary Materials — Supplementary Figure 1: Venn diagram of the number of risk genes in the European population and East Asian population. Supplementary Figure 2: Q − Q plot of the gene-based test computed by MAGMA. Supplementary Figure 3: funnel plot of meta-analysis of rs599839 (PSRC1). Supplementary Figure 4: meta-analysis of the association between rs17465637 (MIA3) and CAD. Supplementary Figure 5: meta-analysis of the association between rs4977574 (CDKN2A/B, ANRIL) and CAD. Supplementary Figure 6: meta-analysis of the association between rs1746048 (CXCL12) and CAD. Supplementary Figure 7: relationship between highly expressed genes in a specific tissue and genetic associations. Supplementary Figure 8: gene expression heat map by hierarchical clustering. Supplementary Figure 9: transcription factor binding site enrichment analysis of CAD susceptibility loci. Supplementary Figure 10: genetic annotation analysis of CAD susceptibility loci. Supplementary Figure 11: histone modification enrichment analysis of CAD susceptibility loci. Supplementary Figure 12: Manhattan plots of SMR tests for association between gene expression and CAD. Supplementary Figure 13: prioritizing genes at GWAS loci using SMR analysis. Supplementary Figure 14: effect sizes of SNPs from GWAS plotted against those for SNPs from eQTL studies. Supplementary Table 1: integrating the population-specific genetic variants reported before and identified by a gene-based test in the present study. Supplementary Table 2: meta-analysis of population-specific genetic variants. Supplementary Table 3: identification of CAD risk genes by using VEGAS. Supplementary Table 4: the results of regulatory element enrichment analysis for CAD in the European population. Supplementary Table 5: the results of regulatory element enrichment analysis for CAD in the Asian population. [file 7036592.f1.zip › CAD_Supplementary_Table1-3.docx]

**Supplementary Table 1. Integrating the population-specific genetic variants reported before and identified by gene-based test in present study.**

| **Gene** | **TopSNP** | **TopSNP-p** |
| --- | --- | --- |
| C9orf53 | rs2069416 | 1.3E-27 |
| CCDC63, MYL2 | rs117607209 | 1.717E-23 |
| RPH3A | rs886477 | 7.674E-13 |
| OAS2, OAS3 | rs3937435 | 2.043E-12 |
| OAS1 | rs886477 | 7.674E-13 |
| OBP2B | rs9328546 | 2.083E-09 |
| BCAS3 | rs142745797 | 4.143E-07 |
| TMEM91, B9D2, TGFB1 | rs4803458 | 6.656E-09 |
| TCF21 | rs2327429 | 1.085E-12 |
| ATXN2 | rs2238153 | 3.274E-08 |
| TAF1A | rs35700460 | 1.827E-09 |
| SH2B3, FAM109A | rs117741012 | 1.365E-08 |
| HYAL1, HYAL2, HYAL3, RASSF1, NAT6, SEMA3B | rs12494414 | 9.619E-07 |
| TMEM115 | rs3774755 | 1.09E-06 |
| PSRC1 | rs599839 | 2.89E-10 |
| PCSK9 | rs11206510 | 9.10E-08 |
| MIA3 | rs17465637 | 1.36E-08 |
| WDR12 | rs6725887 | 1.12E-09 |
| MRAS | rs2306374 | 3.34E-08 |
| PHACTR1 | rs12526453 | 1.15E-09 |
| LPA | rs3798220 | 9.62E-12 |
| CDKN2A/B, ANRIL | rs4977574 | 1.35E-22 |
| CXCL12 | rs1746048 | 2.12E-10 |
| LDLR | rs1122608 | 9.73E-10 |
| MRPS6 | rs9982601 | 4.22E-10 |
| PPAP2B | rs17114036 | 1.43E−08 |
| ANKS1A | rs17609940 | 2.21E−06 |
| ZC3HC1 | rs11556924 | 2.22E−09 |
| ABO | rs579459 | 1.16E−07 |
| CYP17A1, CNNM2, NT5C2 | rs12413409 | 1.47E−06 |
| ZNF259, APOA5-A4-C3-A1 | rs964184 | 8.02E−10 |
| COL4A1, COL4A2 | rs4773144 | 4.15E−07 |
| HHIPL1 | rs2895811 | 2.67E−07 |
| RASD1, SMCR3, PEMT | rs12936587 | 4.89E−07 |
| SMG6, SRR | rs216172 | 6.22E−07 |
| UBE2Z, GIP, ATP5G1, SNF8 | rs46522 | 3.57E−06 |
| RDX, FDX1 | rs10488763 | 2.16E−06 |
| CTSS | rs6587520 | 4.29E−04 |
| WDR11, FGFR2 | rs2257129 | 3.17E−04 |

**Supplementary Table 2. Meta-analysis of population-specific genetic variants**

**A: rs599839 (*PSRC1*)**

| Population | Study | Year | Country | Case/Controls | Male gender (Case/Controls, %) | Age gender (Case/Controls, years) |
| --- | --- | --- | --- | --- | --- | --- |
|  |  |  |  |  |  |  |
| European | Kleber et al.[1] | 2009 | NA | 2506/681 | 75/52 | 64±10/58±12 |
|  | Samani et al.[2] | 2007 | British | 1504/1500 | 79.3/NA | 60.1±8.1/NA |
|  | Samani et al.[2] | 2007 | Germany | 875/1644 | 67.5/NA | 58.1±8.7/NA |
|  | Esparragon et al.[3] | 2012 | Canary etc. | 281/307 | 77.9/73.3 | 55.9/54.6 |
|  | Karvanen et al.[4] | 2009 | Finland etc. | 5613/NA | 74.3/NA | 60.1/NA |
|  | Roder et al.[5] | 2011 | Germany | 40/68 | 70/63 | 25.5/27.6 |
| Asia | Arvind et al.[6] | 2014 | India | 1034/1034 | 77.5/75.9 | 50.13±0.26/50.13±0.25 |
|  | Matsuoka et al.[7] | 2015 | Japan | 1824/2329 | 80.5/44.2 | 64.6±10.3/62.3±11.8 |
|  | Xie et al.[8] | 2011 | China | 1078/2335 | 50.8/66.9 | 60.4±10.3/65.4±10.2 |
|  | Zhou et al. | 2014 | China | 1000/1000 | 63.6/63.6 | 59.69±8.77/59.52±8.54 |

**B:** **rs17465637 (*MIA3*)**

| Population | Study | Year | Country | Case/Controls | Male gender (Case/Controls, %) | Age gender (Case/Controls, years) |
| --- | --- | --- | --- | --- | --- | --- |
|  |  |  |  |  |  |  |
| European | Samani et al.[2] | 2007 | British | 1504/1500 | 79.3/NA | 60.1±8.1/NA |
|  | Samani et al.[2] | 2007 | Germany | 875/1644 | 67.5/NA | 58.1±8.7/NA |
|  | MIGC[9] | 2009 | Italy etc. | 2967/3075 | 78.3/75.4 | 41/43 |
| Asia | Xie et al.[8] | 2011 | China | 1078/2335 | 50.8/66.9 | 60.4±10.3/65.4±10.2 |
|  | Shahid et al.[10] | 2018 | Pakistan | 404/219 | NA | NA |
|  | Li et al.[11] | 2013 | China | 2503/2920 | 66.8/61.1 | 60.6±11.6/47.9±15.6 |
|  | Zhang et al. | 2011 | China | 775/775 | 68/68 | 63.8±9.7/63.7±9.9 |
|  | Hiura et al.[12] | 2008 | Japan | 589/2475 | 86.6/55.5 | 61.3±10.3/65.1±11.0 |

**C:** **rs4977574 (*CDKN2A/B*, *ANRIL*)**

| Population | Study | Year | Country | Case/Controls | Male gender (Case/Controls, %) | Age gender (Case/Controls, years) |
| --- | --- | --- | --- | --- | --- | --- |
|  |  |  |  |  |  |  |
| European | Erdmann et al.[13] | 2009 | Germany | 1222/1298 | 79.7/52.1 | 57.2±8.4/51.2±11.9 |
|  | Erdmann et al.[14] | 2011 | Germany | 875/1644 | 67.5/49.2 | 50.2±7.9/ 62.5±10.1 |
|  | C4D[15] | 2011 | UK etc. | 5720/4381 | NA | NA |
|  | C4D[15] | 2011 | UK | 2704/2887 | NA | 58.8±8.4/NA |
|  | MIGC[9] | 2009 | Italy etc. | 2967/3075 | 78.3/75.4 | 41/43 |
| Asia | Wang et al.[16] | 2014 | China | 2365/2678 | 71.1/69 | 60.8/59.5 |
|  | Schunkert et al.[17] | 2011 | Lebanon | 1524/425 | 77.1/57 | 62±16/NA |
|  | AbdulAzeez et al.[18] | 2016 | Saudi Arabia | 250/252 | 65.2/79.4 | 52.3±13.8/48.1±7.9 |
|  | Hua et al.[19] | 2020 | China | 598/257 | 77.8/63.8 | 61/58 |
|  | Lee et al.[20] | 2014 | China | 925/634 | 80.2/64.4 | 67.6±10.0/ 64.5±10.8 |
|  | Matsuoka et al.[7] | 2015 | Japan | 1824/2329 | 80.5/44.2 | 64.6±10.3/62.3±11.8 |
|  | C4D[15] | 2011 | Pakistan | 4255/4098 | NA | 53.8±10.6/NA |
|  | C4D[15] | 2011 | India | 2741/3696 | NA | 59.3±9.7/NA |

**D: rs1746048 (*CXCL12*)**

| Population | Study | Year | Country | Case/Controls | Male gender (Case/Controls, %) | Age gender (Case/Controls, years) |
| --- | --- | --- | --- | --- | --- | --- |
|  |  |  |  |  |  |  |
| European | C4D[15] | 2011 | UK etc. | 5720/4381 | NA | NA |
|  | C4D[15] | 2011 | UK | 2704/2887 | NA | 58.8±8.4/NA |
|  | MIGC[9] | 2009 | Italy etc. | 2967/3075 | 78.3/75.4 | 41/43 |
|  | C4D[15] | 2011 | UK etc. | 5720/4381 | NA | NA |
|  | C4D[15] | 2011 | UK | 2704/2887 | NA | 58.8±8.4/NA |
|  | Mehta et al.[21] | 2011 | America | 933/468 | NA | NA |
| Asia | C4D[15] | 2011 | Pakistan | 4255/4098 | NA | 53.8±10.6/NA |
|  | C4D[15] | 2011 | India | 2741/3696 | NA | 59.3±9.7/NA |
|  | Shahid et al.[10] | 2018 | Pakistan | 404/219 | NA | NA |
|  | Wang et al.[16] | 2014 | China | 2365/2678 | 71.1/69 | 60.8/59.5 |
|  | Huang et al.[22] | 2013 | China | 434/358 | 70.5/ | 61.98±9.49/58.65±9.36 |
|  | C4D[15] | 2011 | Pakistan | 4255/4098 | NA | 53.8±10.6/NA |
|  | C4D[15] | 2011 | India | 2741/3696 | NA | 59.3±9.7/NA |

MIGC: Myocardial Infarction Genetics Consortium

C4D: The Coronary Artery Disease (C4D) Genetics Consortium

**Supplementary Table 3. Identification of CAD risk genes by using VEGAS.**

| **Population** | **Chr** | **Gene** | **nSNPs** | **Test** | **Pvalue** | **TopSNP** | **TopSNP-p** |
| --- | --- | --- | --- | --- | --- | --- | --- |
| **European** | 9 | CDKN2B-AS1 | 225 | 7618.96 | 1E-06 | rs1333045 | 4.63E-32 |
|  | 6 | PHACTR1 | 689 | 3638.27 | 1E-06 | rs4714955 | 6.03E-12 |
|  | 9 | CDKN2B | 93 | 3630.71 | 1E-06 | rs10738604 | 2.27E-30 |
|  | 9 | CDKN2A | 101 | 3124.27 | 1E-06 | rs10738604 | 2.27E-30 |
|  | 15 | ADAMTS7 | 66 | 1096.68 | 1E-06 | rs2219939 | 1.21E-09 |
|  | 12 | CUX2 | 113 | 1051.29 | 2E-06 | rs4766453 | 1.8E-06 |
|  | 9 | C9orf53 | 67 | 1036.79 | 1E-06 | rs2069416 | 1.3E-27 |
|  | 3 | MRAS | 81 | 873.402 | 2E-06 | rs2306374 | 3.34E-08 |
|  | 15 | LOC646938 | 43 | 740.386 | 1E-06 | rs2219939 | 1.21E-09 |
|  | 2 | ICA1L | 59 | 677.611 | 1E-06 | rs3845800 | 4.13E-11 |
|  | 2 | CARF | 48 | 590.357 | 1E-06 | rs2351524 | 1.76E-11 |
|  | 2 | WDR12 | 34 | 565.069 | 1E-06 | rs3845800 | 4.13E-11 |
| **Asian** | 9 | CDKN2B-AS1 | 450 | 10569.5 | 1E-06 | rs10757274 | 6.331E-27 |
|  | 12 | LOC100131138 | 138 | 7747.81 | 1E-06 | rs117607209 | 1.717E-23 |
|  | 12 | CCDC63 | 206 | 7691.37 | 1E-06 | rs117607209 | 1.717E-23 |
|  | 12 | MYL2 | 134 | 7625.37 | 1E-06 | rs117607209 | 1.717E-23 |
|  | 12 | CUX2 | 360 | 6077.64 | 1E-06 | rs79105258 | 6.547E-32 |
|  | 9 | ABO | 546 | 5884.47 | 1E-06 | rs9411378 | 1.89E-10 |
|  | 12 | RPH3A | 403 | 5726.44 | 1E-06 | rs886477 | 7.674E-13 |
|  | 15 | ADAMTS7 | 401 | 5167.9 | 1E-06 | rs28455815 | 1.241E-08 |
|  | 10 | CNNM2 | 603 | 5016.62 | 2E-06 | rs11191559 | 2.165E-08 |
|  | 11 | FDX1 | 367 | 4670.64 | 1E-06 | rs1848599 | 3.866E-08 |
|  | 12 | OAS3 | 336 | 4020.37 | 2E-06 | rs3937435 | 2.043E-12 |
|  | 12 | OAS1 | 259 | 3894.35 | 1E-06 | rs886477 | 7.674E-13 |
|  | 9 | CDKN2B | 198 | 3832.05 | 1E-06 | rs2518723 | 3.421E-15 |
|  | 12 | OAS2 | 296 | 3139.87 | 1E-06 | rs3937435 | 2.043E-12 |
|  | 9 | OBP2B | 378 | 3129.9 | 1E-06 | rs9328546 | 2.083E-09 |
|  | 12 | MIR6760 | 193 | 3050.59 | 1E-06 | rs79105258 | 6.547E-32 |
|  | 19 | TMEM91 | 184 | 3040.98 | 1E-06 | rs4803458 | 6.656E-09 |
|  | 9 | CDKN2A | 209 | 3015.64 | 1E-06 | rs2518723 | 3.421E-15 |
|  | 17 | BCAS3 | 429 | 2961.01 | 2E-06 | rs142745797 | 4.143E-07 |
|  | 1 | MIA3 | 283 | 2937.57 | 1E-06 | rs67180937 | 3.179E-10 |
|  | 6 | LINC01312 | 210 | 2836.87 | 1E-06 | rs2327429 | 1.085E-12 |
|  | 11 | MIR4693 | 243 | 2627.51 | 1E-06 | rs11601633 | 3.341E-09 |
|  | 19 | B9D2 | 153 | 2423.92 | 2E-06 | rs4803458 | 6.656E-09 |
|  | 19 | TGFB1 | 166 | 2183.65 | 1E-06 | rs4803458 | 6.656E-09 |
|  | 6 | TCF21 | 168 | 2111.74 | 1E-06 | rs2327429 | 1.085E-12 |
|  | 12 | ATXN2 | 161 | 2045.8 | 1E-06 | rs2238153 | 3.274E-08 |
|  | 1 | TAF1A | 300 | 1954 | 2E-06 | rs35700460 | 1.827E-09 |
|  | 1 | LOC100506161 | 224 | 1925.66 | 2E-06 | rs35700460 | 1.827E-09 |
|  | 12 | FAM109A | 162 | 1892.17 | 1E-06 | rs117741012 | 1.365E-08 |
|  | 15 | LOC646938 | 150 | 1783.63 | 1E-06 | rs1825084 | 3.285E-08 |
|  | 3 | HYAL1 | 113 | 1584.99 | 2E-06 | rs12494414 | 9.619E-07 |
|  | 12 | SH2B3 | 118 | 1497.71 | 1E-06 | rs117741012 | 1.365E-08 |
|  | 3 | HYAL3 | 111 | 1493.38 | 1E-06 | rs12494414 | 9.619E-07 |
|  | 3 | LSMEM2 | 113 | 1471.6 | 2E-06 | rs12494414 | 9.619E-07 |
|  | 3 | IFRD2 | 112 | 1469.57 | 2E-06 | rs12494414 | 9.619E-07 |
|  | 3 | RASSF1 | 115 | 1466.91 | 2E-06 | rs12494414 | 9.619E-07 |
|  | 3 | NAT6 | 108 | 1448.7 | 1E-06 | rs12494414 | 9.619E-07 |
|  | 3 | HYAL2 | 110 | 1432.29 | 2E-06 | rs12494414 | 9.619E-07 |
|  | 3 | RASSF1-AS1 | 106 | 1351.91 | 1E-06 | rs3774755 | 1.09E-06 |
|  | 3 | SEMA3B | 106 | 1344.5 | 2E-06 | rs12494414 | 9.619E-07 |
|  | 3 | SEMA3B-AS1 | 100 | 1266.29 | 2E-06 | rs12494414 | 9.619E-07 |
|  | 3 | TMEM115 | 109 | 1167.9 | 2E-06 | rs3774755 | 1.09E-06 |

**Reference**

1. M. E. Kleber, W. Renner, T. B. Grammer, P. Linsel-Nitschke, B. O. Boehm, B. R. Winkelmann, P. Bugert, M. M. Hoffmann and W. Marz, "Association of the single nucleotide polymorphism rs599839 in the vicinity of the sortilin 1 gene with LDL and triglyceride metabolism, coronary heart disease and myocardial infarction. The Ludwigshafen Risk and Cardiovascular Health Study," *Atherosclerosis*, vol. 209, no. 2, pp. 492-497, 2010.

2. N. J. Samani, J. Erdmann, A. S. Hall, C. Hengstenberg, M. Mangino, B. Mayer, R. J. Dixon, T. Meitinger, P. Braund, H. E. Wichmann, J. H. Barrett, I. R. Konig, S. E. Stevens, S. Szymczak, D. A. Tregouet, M. M. Iles, F. Pahlke, H. Pollard, W. Lieb, F. Cambien, M. Fischer, W. Ouwehand, S. Blankenberg, A. J. Balmforth, A. Baessler, S. G. Ball, T. M. Strom, I. Braenne, C. Gieger, P. Deloukas, M. D. Tobin, A. Ziegler, J. R. Thompson, H. Schunkert, Wtccc and C. the Cardiogenics, "Genomewide association analysis of coronary artery disease," *N Engl J Med*, vol. 357, no. 5, pp. 443-453, 2007.

3. F. R. Esparragon, O. Companioni, M. G. Bello, N. B. Rios and J. C. R. Perez, "Replication of relevant SNPs associated with cardiovascular disease susceptibility obtained from GWAs in a case-control study in a Canarian population," *Disease Markers*, vol. 32, no. 4, pp. 231-239, 2012.

4. J. Karvanen, K. Silander, F. Kee, L. Tiret, V. Salomaa, K. Kuulasmaa, P. G. Wiklund, J. Virtamo, O. Saarela, C. Perret, M. Perola, L. Peltonen, F. Cambien, J. Erdmann, N. J. Samani, H. Schunkert, A. Evans and M. Project, "The impact of newly identified loci on coronary heart disease, stroke and total mortality in the MORGAM prospective cohorts," *Genet Epidemiol*, vol. 33, no. 3, pp. 237-246, 2009.

5. C. Roder, V. Peters, H. Kasuya, T. Nishizawa, Y. Takehara, D. Berg, C. Schulte, N. Khan, M. Tatagiba and B. Krischek, "Common genetic polymorphisms in moyamoya and atherosclerotic disease in Europeans," *Childs Nerv Syst*, vol. 27, no. 2, pp. 245-252, 2011.

6. P. Arvind, J. Nair, S. Jambunathan, V. V. Kakkar and J. Shanker, "CELSR2-PSRC1-SORT1 gene expression and association with coronary artery disease and plasma lipid levels in an Asian Indian cohort," *Journal of Cardiology*, vol. 64, no. 5-6, pp. 339-346, 2014.

7. R. Matsuoka, S. Abe, F. Tokoro, M. Arai, T. Noda, S. Watanabe, H. Horibe, T. Fujimaki, M. Oguri, K. Kato, S. Minatoguchi and Y. Yamada, "Association of six genetic variants with myocardial infarction," *Int J Mol Med*, vol. 35, no. 5, pp. 1451-1459, 2015.

8. F. Xie, X. Chu, H. Wu, W. Sun, M. Shen, L. Yang, Y. Wang, Y. Wang, J. Shi and W. Huang, "Replication of putative susceptibility loci from genome-wide association studies associated with coronary atherosclerosis in Chinese Han population," *PLoS One*, vol. 6, no. 6, pp. e20833, 2011.

9. C. Myocardial Infarction Genetics, S. Kathiresan, B. F. Voight, S. Purcell, K. Musunuru, D. Ardissino, P. M. Mannucci, S. Anand, J. C. Engert, N. J. Samani, H. Schunkert, J. Erdmann, M. P. Reilly, D. J. Rader, T. Morgan, J. A. Spertus, M. Stoll, D. Girelli, P. P. McKeown, C. C. Patterson, D. S. Siscovick, C. J. O'Donnell, R. Elosua, L. Peltonen, V. Salomaa, S. M. Schwartz, O. Melander, D. Altshuler, D. Ardissino, P. A. Merlini, C. Berzuini, L. Bernardinelli, F. Peyvandi, M. Tubaro, P. Celli, M. Ferrario, R. Fetiveau, N. Marziliano, G. Casari, M. Galli, F. Ribichini, M. Rossi, F. Bernardi, P. Zonzin, A. Piazza, P. M. Mannucci, S. M. Schwartz, D. S. Siscovick, J. Yee, Y. Friedlander, R. Elosua, J. Marrugat, G. Lucas, I. Subirana, J. Sala, R. Ramos, S. Kathiresan, J. B. Meigs, G. Williams, D. M. Nathan, C. A. MacRae, C. J. O'Donnell, V. Salomaa, A. S. Havulinna, L. Peltonen, O. Melander, G. Berglund, B. F. Voight, S. Kathiresan, J. N. Hirschhorn, R. Asselta, S. Duga, M. Spreafico, K. Musunuru, M. J. Daly, S. Purcell, B. F. Voight, S. Purcell, J. Nemesh, J. M. Korn, S. A. McCarroll, S. M. Schwartz, J. Yee, S. Kathiresan, G. Lucas, I. Subirana, R. Elosua, A. Surti, C. Guiducci, L. Gianniny, D. Mirel, M. Parkin, N. Burtt, S. B. Gabriel, N. J. Samani, J. R. Thompson, P. S. Braund, B. J. Wright, A. J. Balmforth, S. G. Ball, A. Hall, C. Wellcome Trust Case Control, H. Schunkert, J. Erdmann, P. Linsel-Nitschke, W. Lieb, A. Ziegler, I. Konig, C. Hengstenberg, M. Fischer, K. Stark, A. Grosshennig, M. Preuss, H. E. Wichmann, S. Schreiber, H. Schunkert, N. J. Samani, J. Erdmann, W. Ouwehand, C. Hengstenberg, P. Deloukas, M. Scholz, F. Cambien, M. P. Reilly, M. Li, Z. Chen, R. Wilensky, W. Matthai, A. Qasim, H. H. Hakonarson, J. Devaney, M. S. Burnett, A. D. Pichard, K. M. Kent, L. Satler, J. M. Lindsay, R. Waksman, C. W. Knouff, D. M. Waterworth, M. C. Walker, V. Mooser, S. E. Epstein, D. J. Rader, T. Scheffold, K. Berger, M. Stoll, A. Huge, D. Girelli, N. Martinelli, O. Olivieri, R. Corrocher, T. Morgan, J. A. Spertus, P. McKeown, C. C. Patterson, H. Schunkert, E. Erdmann, P. Linsel-Nitschke, W. Lieb, A. Ziegler, I. R. Konig, C. Hengstenberg, M. Fischer, K. Stark, A. Grosshennig, M. Preuss, H. E. Wichmann, S. Schreiber, H. Holm, G. Thorleifsson, U. Thorsteinsdottir, K. Stefansson, J. C. Engert, R. Do, C. Xie, S. Anand, S. Kathiresan, D. Ardissino, P. M. Mannucci, D. Siscovick, C. J. O'Donnell, N. J. Samani, O. Melander, R. Elosua, L. Peltonen, V. Salomaa, S. M. Schwartz and D. Altshuler, "Genome-wide association of early-onset myocardial infarction with single nucleotide polymorphisms and copy number variants," *Nat Genet*, vol. 41, no. 3, pp. 334-341, 2009.

10. S. U. Shahid, N. A. Shabana, A. Rehman and S. Humphries, "GWAS implicated risk variants in different genes contribute additively to increase the risk of coronary artery disease (CAD) in the Pakistani subjects," *Lipids in Health and Disease*, vol. 17, 2018.

11. X. C. Li, Y. F. Huang, D. Yin, D. Wang, C. Q. Xu, F. Wang, Q. Yang, X. J. Wang, S. S. Li, S. S. Chen, X. Xiong, Y. Huang, Y. Y. Zhao, L. Wang, X. Zhu, Z. H. Su, B. S. Zhou, Y. T. Zhang, L. F. Wang, L. Chang, C. P. Xu, H. Li, T. Ke, X. Ren, X. Cheng, Y. Z. Yang, Y. H. Liao, X. Tu and Q. K. Wang, "Meta-analysis identifies robust association between SNP rs17465637 in MIA3 on chromosome 1q41 and coronary artery disease," *Atherosclerosis*, vol. 231, no. 1, pp. 136-140, 2013.

12. Y. Hiura, Y. Fukushima, M. Yuno, H. Sawamura, Y. Kokubo, T. Okamura, H. Tomoike, Y. Goto, H. Nonogi, R. Takahashi and N. Iwai, "Validation of the association of genetic variants on chromosome 9p21 and 1q41 with myocardial infarction in a Japanese population," *Circ J*, vol. 72, no. 8, pp. 1213-1217, 2008.

13. J. Erdmann, A. Grosshennig, P. S. Braund, I. R. Konig, C. Hengstenberg, A. S. Hall, P. Linsel-Nitschke, S. Kathiresan, B. Wright, D. A. Tregouet, F. Cambien, P. Bruse, Z. Aherrahrou, A. K. Wagner, K. Stark, S. M. Schwartz, V. Salomaa, R. Elosua, O. Melander, B. F. Voight, C. J. O'Donnell, L. Peltonen, D. S. Siscovick, D. Altshuler, P. A. Merlini, F. Peyvandi, L. Bernardinelli, D. Ardissino, A. Schillert, S. Blankenberg, T. Zeller, P. Wild, D. F. Schwarz, L. Tiret, C. Perret, S. Schreiber, N. E. El Mokhtari, A. Schafer, W. Marz, W. Renner, P. Bugert, H. Kluter, J. Schrezenmeir, D. Rubin, S. G. Ball, A. J. Balmforth, H. E. Wichmann, T. Meitinger, M. Fischer, C. Meisinger, J. Baumert, A. Peters, W. H. Ouwehand, T. Italian Atherosclerosis, G. Vascular Biology Working, C. Myocardial Infarction Genetics, C. Wellcome Trust Case Control, C. Cardiogenics, P. Deloukas, J. R. Thompson, A. Ziegler, N. J. Samani and H. Schunkert, "New susceptibility locus for coronary artery disease on chromosome 3q22.3," *Nat Genet*, vol. 41, no. 3, pp. 280-282, 2009.

14. J. Erdmann, C. Willenborg, J. Nahrstaedt, M. Preuss, I. R. Konig, J. Baumert, P. Linsel-Nitschke, C. Gieger, S. Tennstedt, P. Belcredi, Z. Aherrahrou, N. Klopp, C. Loley, K. Stark, C. Hengstenberg, P. Bruse, J. Freyer, A. K. Wagner, A. Medack, W. Lieb, A. Grosshennig, H. B. Sager, A. Reinhardt, A. Schafer, S. Schreiber, N. E. El Mokhtari, D. Raaz-Schrauder, T. Illig, C. D. Garlichs, A. B. Ekici, A. Reis, J. Schrezenmeir, D. Rubin, A. Ziegler, H. E. Wichmann, A. Doering, C. Meisinger, T. Meitinger, A. Peters and H. Schunkert, "Genome-wide association study identifies a new locus for coronary artery disease on chromosome 10p11.23," *Eur Heart J*, vol. 32, no. 2, pp. 158-168, 2011.

15. C. Coronary Artery Disease Genetics, "A genome-wide association study in Europeans and South Asians identifies five new loci for coronary artery disease," *Nat Genet*, vol. 43, no. 4, pp. 339-344, 2011.

16. Y. Q. Wang, L. F. Wang, X. Liu, Y. Z. Zhang, L. P. Yu, F. Zhang, L. S. Liu, J. Cai, X. C. Yang and X. Y. Wang, "Genetic Variants Associated with Myocardial Infarction and the Risk Factors in Chinese Population," *Plos One*, vol. 9, no. 1, 2014.

17. H. Schunkert, I. R. Konig, S. Kathiresan, M. P. Reilly, T. L. Assimes, H. Holm, M. Preuss, A. F. Stewart, M. Barbalic, C. Gieger, D. Absher, Z. Aherrahrou, H. Allayee, D. Altshuler, S. S. Anand, K. Andersen, J. L. Anderson, D. Ardissino, S. G. Ball, A. J. Balmforth, T. A. Barnes, D. M. Becker, L. C. Becker, K. Berger, J. C. Bis, S. M. Boekholdt, E. Boerwinkle, P. S. Braund, M. J. Brown, M. S. Burnett, I. Buysschaert, Cardiogenics, J. F. Carlquist, L. Chen, S. Cichon, V. Codd, R. W. Davies, G. Dedoussis, A. Dehghan, S. Demissie, J. M. Devaney, P. Diemert, R. Do, A. Doering, S. Eifert, N. E. Mokhtari, S. G. Ellis, R. Elosua, J. C. Engert, S. E. Epstein, U. de Faire, M. Fischer, A. R. Folsom, J. Freyer, B. Gigante, D. Girelli, S. Gretarsdottir, V. Gudnason, J. R. Gulcher, E. Halperin, N. Hammond, S. L. Hazen, A. Hofman, B. D. Horne, T. Illig, C. Iribarren, G. T. Jones, J. W. Jukema, M. A. Kaiser, L. M. Kaplan, J. J. Kastelein, K. T. Khaw, J. W. Knowles, G. Kolovou, A. Kong, R. Laaksonen, D. Lambrechts, K. Leander, G. Lettre, M. Li, W. Lieb, C. Loley, A. J. Lotery, P. M. Mannucci, S. Maouche, N. Martinelli, P. P. McKeown, C. Meisinger, T. Meitinger, O. Melander, P. A. Merlini, V. Mooser, T. Morgan, T. W. Muhleisen, J. B. Muhlestein, T. Munzel, K. Musunuru, J. Nahrstaedt, C. P. Nelson, M. M. Nothen, O. Olivieri, R. S. Patel, C. C. Patterson, A. Peters, F. Peyvandi, L. Qu, A. A. Quyyumi, D. J. Rader, L. S. Rallidis, C. Rice, F. R. Rosendaal, D. Rubin, V. Salomaa, M. L. Sampietro, M. S. Sandhu, E. Schadt, A. Schafer, A. Schillert, S. Schreiber, J. Schrezenmeir, S. M. Schwartz, D. S. Siscovick, M. Sivananthan, S. Sivapalaratnam, A. Smith, T. B. Smith, J. D. Snoep, N. Soranzo, J. A. Spertus, K. Stark, K. Stirrups, M. Stoll, W. H. Tang, S. Tennstedt, G. Thorgeirsson, G. Thorleifsson, M. Tomaszewski, A. G. Uitterlinden, A. M. van Rij, B. F. Voight, N. J. Wareham, G. A. Wells, H. E. Wichmann, P. S. Wild, C. Willenborg, J. C. Witteman, B. J. Wright, S. Ye, T. Zeller, A. Ziegler, F. Cambien, A. H. Goodall, L. A. Cupples, T. Quertermous, W. Marz, C. Hengstenberg, S. Blankenberg, W. H. Ouwehand, A. S. Hall, P. Deloukas, J. R. Thompson, K. Stefansson, R. Roberts, U. Thorsteinsdottir, C. J. O'Donnell, R. McPherson, J. Erdmann, C. A. Consortium and N. J. Samani, "Large-scale association analysis identifies 13 new susceptibility loci for coronary artery disease," *Nat Genet*, vol. 43, no. 4, pp. 333-338, 2011.

18. S. AbdulAzeez, A. N. Al-Nafie, A. Al-Shehri, J. F. Borgio, E. V. Baranova, M. S. Al-Madan, R. A. Al-Ali, F. Al-Muhanna, A. Al-Ali, M. Al-Mansori, M. F. Ibrahim, F. W. Asselbergs, B. Keating, B. P. C. Koeleman and A. K. Al-Ali, "Intronic Polymorphisms in the CDKN2B-AS1 Gene Are Strongly Associated with the Risk of Myocardial Infarction and Coronary Artery Disease in the Saudi Population," *International Journal of Molecular Sciences*, vol. 17, no. 3, 2016.

19. L. Hua, J. X. Yuan, S. He, C. H. Zhao, Q. W. Jia, J. Zhang, F. H. An, Z. H. Chen, L. H. Li, L. S. Wang, W. Z. Ma, G. X. Xu and E. Z. Jia, "Analysis on the polymorphisms of site RS4977574, and RS1333045 in region 9p21 and the susceptibility of coronary heart disease in Chinese population," *Bmc Medical Genetics*, vol. 21, no. 1, 2020.

20. I. T. Lee, M. O. Goodarzi, W. J. Lee, J. I. Rotter, Y. D. Chen, K. W. Liang, W. L. Lee and W. H. Sheu, "The chromosome 9p21 variant not predicting long-term cardiovascular mortality in Chinese with established coronary artery disease: an eleven-year follow-up study," *Biomed Res Int*, vol. 2014, pp. 626907, 2014.

21. N. N. Mehta, M. Li, D. William, A. V. Khera, S. DerOhannessian, L. Qu, J. F. Ferguson, C. McLaughlin, L. H. Shaikh, R. Shah, P. N. Patel, J. P. Bradfield, J. He, I. M. Stylianou, H. Hakonarson, D. J. Rader and M. P. Reilly, "The novel atherosclerosis locus at 10q11 regulates plasma CXCL12 levels," *Eur Heart J*, vol. 32, no. 8, pp. 963-971, 2011.

22. Y. Huang, J. Q. Zhou, H. D. Ye, L. M. Xu, Y. P. Le, X. Yang, W. F. Xu, X. Y. Huang, J. F. Lian and S. W. Duan, "Relationship between chemokine (C-X-C motif) ligand 12 gene variant (rs1746048) and coronary heart disease: Case-control study and meta-analysis," *Gene*, vol. 521, no. 1, pp. 38-44, 2013.
